# HAPPILEE: HAPPE In Low Electrode Electroencephalography, a standardized pre-processing software for lower density recordings

**DOI:** 10.1016/j.neuroimage.2022.119390

**Published:** 2022-10-15

**Authors:** K.L. Lopez, A.D. Monachino, S. Morales, S.C. Leach, M.E. Bowers, L.J. Gabard-Durnam

**Affiliations:** a Northeastern University, 360 Huntington Ave, Boston, MA, United States; b University of Maryland, College Park, MD, United States

**Keywords:** Electroencephalography, Processing pipeline, Low-density EEG, HAPPILEE, Automated pre-processing

## Abstract

Lower-density Electroencephalography (EEG) recordings (from 1 to approximately 32 electrodes) are widely-used in research and clinical practice and enable scalable brain function measurement across a variety of settings and populations. Though a number of automated pipelines have recently been proposed to standardize and optimize EEG pre-processing for high-density systems with state-of-the-art methods, few solutions have emerged that are compatible with lower-density systems. However, lower-density data often include long recording times and/or large sample sizes that would benefit from similar standardization and automation with contemporary methods. To address this need, we propose the HAPPE In Low Electrode Electroencephalography (HAPPILEE) pipeline as a standardized, automated pipeline optimized for EEG recordings with lower density channel layouts of any size. HAPPILEE processes task-free (e.g., resting-state) and task-related EEG (including event-related potential data by interfacing with the HAPPE+ER pipeline), from raw files through a series of processing steps including filtering, line noise reduction, bad channel detection, artifact correction from continuous data, segmentation, and bad segment rejection that have all been optimized for lower density data. HAPPILEE also includes post-processing reports of data and pipeline quality metrics to facilitate the evaluation and reporting of data quality and processing-related changes to the data in a standardized manner. Here the HAPPILEE steps and their optimization with both recorded and simulated EEG data are described. HAPPILEE's performance is then compared relative to other artifact correction and rejection strategies. The HAPPILEE pipeline is freely available as part of HAPPE 2.0 software under the terms of the GNU General Public License at: https://github.com/PINE-Lab/HAPPE.

## 1 Introduction

Electroencephalography (EEG) recordings are a useful and noninvasive tool for interrogating human brain function across the lifespan. Advancements in neuroimaging technology and computer science have allowed for rich data collection in laboratories through the use of high-density channel layouts, but it is not always feasible or optimal to rely on these dense layouts. Low-density channel layouts (fewer than approximately 32 channels) continue to be heavily used, particularly with clinical populations, both in clinical research (Brito et al., 2019, 2016; Gu et al., 2018; Pellinen et al., 2020; van den Munckhof et al., 2018) and diagnostic testing (Aeby et al., 2021; Cassani et al., 2017; Paul et al., 2019; Tiwari et al., 2017), as well as in low-resource areas (Kariuki et al., 2016; Siddiqi et al., 2015; Sokolov et al., 2020; Williams et al., 2019). A low-density EEG approach also provides the flexibility for researchers to travel to participants for testing in natural contexts (e.g. school-based or home-based studies, Troller-Renfree et al., 2021) or in the event that participants cannot come to the lab. Low-density EEG will also be instrumental in future research, given the current momentum towards large-scale neuroscience studies that achieve community implementation and the focus on precision medicine through brain-based biomarkers (e.g., potential EEG-based screening for Autism Spectrum Disorder at well-child doctor's visits), where high-density recordings may be neither practical nor necessary. Indeed, a number of wearable, ultra-low-cost, low-density EEG hardware solutions are emerging in industry to facilitate such measurement. A key impediment to the use of low-density EEG in these contexts is the fact that the raw EEG signal is contaminated by both environmental and physiological artifacts. Up to this point, researcher selection of uncontaminated EEG data has been standard practice, but even with low-density data, this method is time-consuming, subjective, and does not allow for the efficient processing of a large number of data sets. Low-density EEG collected in clinical contexts that can span hours may also preclude manual inspection due to recording length. As a result, there remains a current and growing need for software that standardizes and automates the processing and removal of artifacts in low-density EEG data.

There is now an extensive collection of automated EEG processing pipelines (e.g., Andersen 2018; APP, da Cruz et al. 2018; MADE, Debnath et al. 2020; EEG-IP-L, Desjardins et al. 2021; HAPPE, Gabard-Durnam et al. 2018; Hatz et al. 2015; FASTER, Nolan et al. 2010; Automagic, Pedroni et al. 2019; EPOS, Rodrigues et al. 2020). However, their reliance on independent component analysis (ICA) to segregate and correct artifacts makes them unsustainable for low-density data, as the limited number of channels provides insufficient independent components for robust artifact isolation. Many of these pipelines also use standard deviation-dependent approaches to identify outlier data or channels as artifact-contaminated. These approaches may require modification to scale down to low-density setups with few channels. Other software tools built into these fully-automated pipelines to aid in different stages of artifact detection are most effective when used with high-density data or have not been validated in low-density data (PREP, Bigdely-Shamlo et al. 2015a; SASICA, Chaumon et al. 2015; Adjusted-ADJUST, Leach et al. 2020; ADJUST, Mognon et al. 2011; ASR, Mullen et al. 2013; MARA, Winkler et al. 2014). A recent automated mega-analysis by Bigdely-Shamlo et al. (2020) introduced a pipeline that supports both high-density and low-density data, but only at the upper bound of low-density channel layouts. Specifically, they tested their pipeline using data sets ranging from a Neuroscan 30-channel headset (Compumedics Neuroscan) to a Biosemi 256-channel headset (Biosemi B.V.), but found that the density of the headset accounted for variability in the channel amplitudes across datasets after processing. Several pipelines automate the processing of strictly low-density data. Of these options, some are made specifically for a particular population (Cassani et al., 2017) or acquisition systems (e.g., James Long EEG Analysis System software, Whedon et al. 2020). Others use independent component analysis to correct artifacts (Hajra et al., 2020), which cannot support many low-density setups. Still others offer only artifact-rejection approaches (e.g., channel and/or segment rejection), some of which target only specific classes of artifact (e.g., eye-blink artifacts), and these artifact-rejection methods can cause significant data loss without artifact-correction in continuous data first (e.g., EEG Analysis System software (James Long Company), MINIMADE, Troller-Renfree et al. 2021). Thus, there remains a need for standardized processing solutions to serve the range of low-density EEG configurations in use and the range of artifacts that occur in EEG data.

**Fig. 1:**
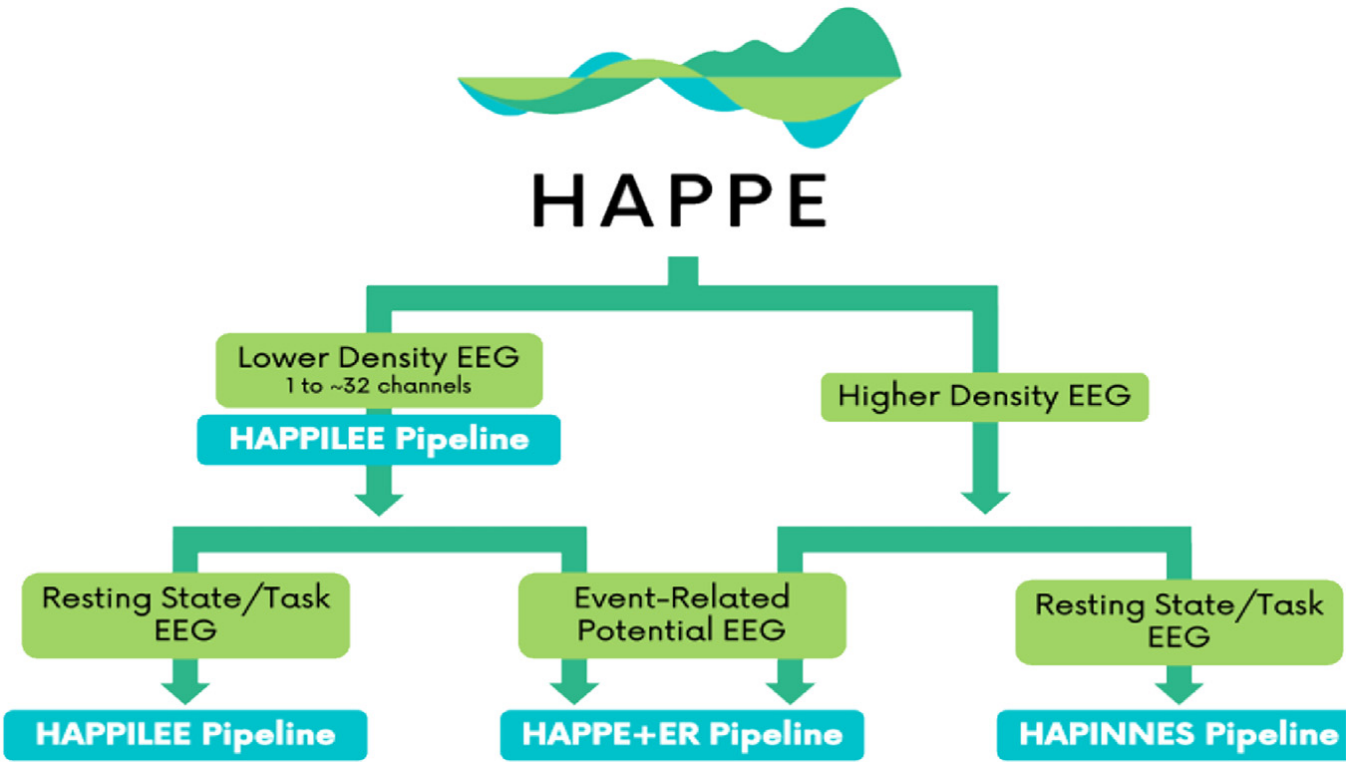
Schematic illustrating the various pipelines within the HAPPE software package.

**Fig. 2:**
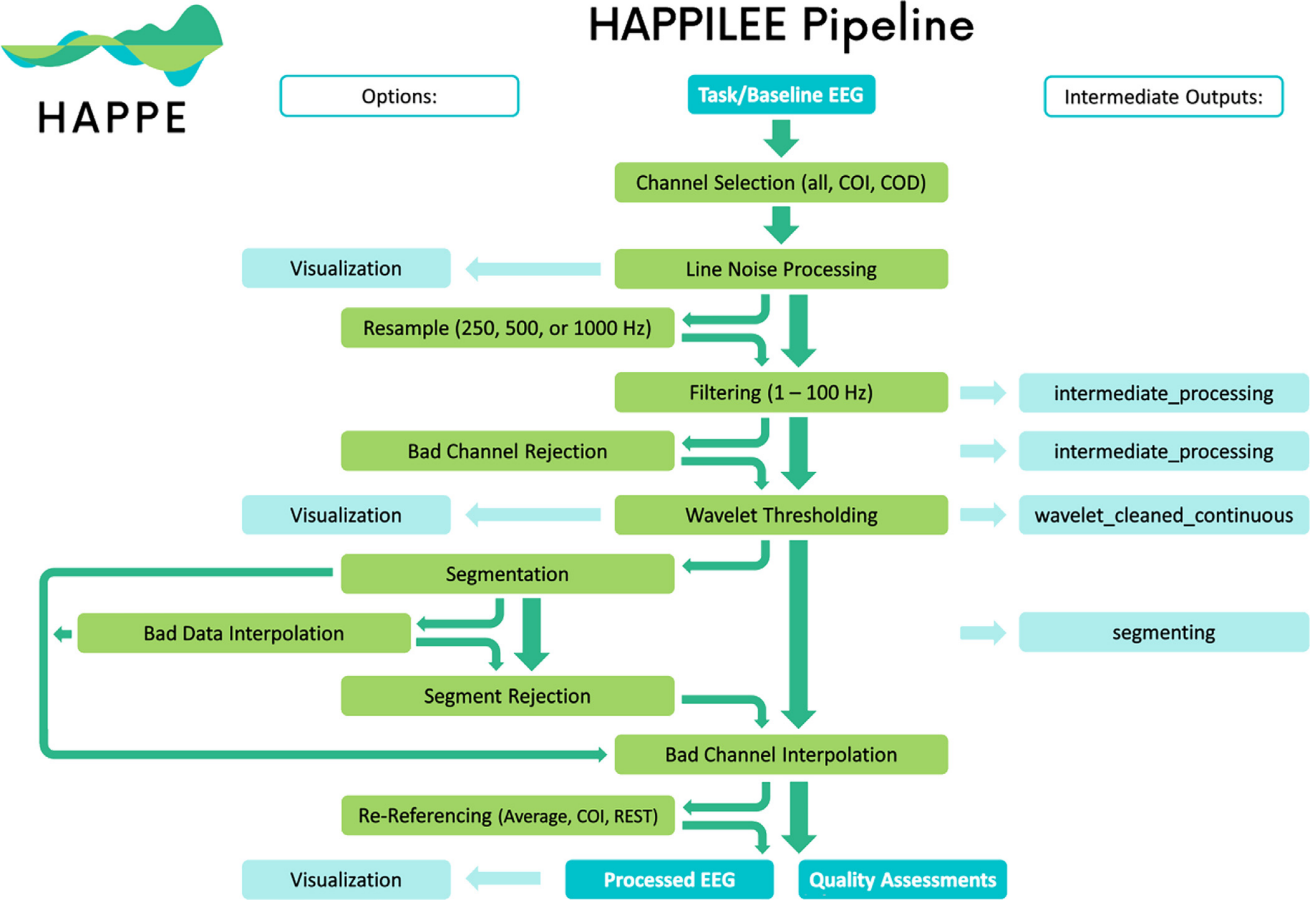
Schematic illustrating the HAPPILEE pipeline's processing steps. The intermediate output EEG files are indicated by the suffix added after that specific processing step in the light blue boxes. The user options for resampling, segmentation, bad data interpolation, segment rejection, and re-referencing steps and visualizing several steps in HAPPILEE with the semi-automated setting are also indicated.

To address this need, we propose a novel pipeline for low-density EEG data (fewer than 32 channels) called HAPPILEE (Harvard Automated Pre-processing Pipeline Including Low-Electrode Encephalography). We apply contemporary approaches to optimize line noise reduction, bad channel detection, artifact correction from continuous data, segmentation, and bad segment rejection methods to suit low density datasets. HAPPILEE is embedded in the HAPPE software package, which facilitates pre-processing for a variety of analyses and data types (Fig. 1). To facilitate ERP analyses on low density data, HAPPILEE interfaces with the HAPPE+ER pipeline within HAPPE (Monachino et al., 2021). Because modifications for ERP analyses are not density-dependent (with the exception of the optional bad channel detection step, which uses HAPPILEE optimization criteria for low density ERP analyses), specific details on the optimization of this software for ERP analyses can be found in Monachino et al. (2021). The following sections of this manuscript describe HAPPILEE's processing steps and outputs, assess optimization of these steps for low-density EEG, and demonstrate HAPPILEE's effectiveness with a low-density developmental EEG dataset and simulated EEG signals relative to other pre-processing approaches (see Fig. 2 for full pipeline schematic).

### 1.1 Optimization dataset

The various steps of the HAPPILEE automated pipeline were optimized using a subset of developmental EEG files from the Bucharest Early Intervention Project (BEIP) (for full study design, see Zeanah et al. 2003). The EEG files contributing to this example dataset may be freely assessed at: https://zenodo.org/record/5,088,346 (Lopez et al., 2021). We selected the BEIP dataset as its study design facilitated testing HAPPILEE on EEG data from children across a range of caregiving conditions, behavioral/clinical phenotypes, and ages (Zeanah et al., 2009). The optimization dataset includes resting-state EEG from three groups of children living in Romania starting in 2001. The first group, referred to as the Care as Usual Group (CAUG), is composed of children living across six institutionalized care facilities throughout Bucharest, Romania. The second group is the Foster Care Group (FCG), which is composed of children who were removed from these institutions through random assignment and placed in a foster care intervention. The final group is the Never Institutionalized Group (NIG), made up of a community sample of children living with their biological families who have never been placed in institutionalized care or foster care. We selected a subset of thirty EEG files across the three groups from the greater dataset (CAUG, *n* = 8; FCG, *n* = 8, NIG, *n* = 14). The average age at the start of the baseline assessment across the three groups was 17.40 months, with a range of 6.28–29.98 months (averages per group: CAUG=18.30; FCG=16.21; NIG=17.58). The resting-state EEG for all children was recorded with the James Long system from twelve scalp sites (F3, F4, Fz, C3, C4, P3, P4, Pz, O1, O2, T7 and T8) using a lycra Electro-Cap (Electro-Cap International Inc., Eaton, OH) with sewn-in tin electrodes (the two mastoid sites, M1 and M2, were removed due to poor recording quality across the dataset).

### 1.2 HAPPILEE data inputs

HAPPILEE accommodates multiple types of EEG files with different acquisition layouts as inputs. A single run will support only a single file type across files, specified by the user. For .set formated files, the correct channel locations should be pre-set and embedded in the file (e.g. by loading it into EEGLAB and confirming the correct locations) prior to running through HAPPILEE. When running .mat formated files, you must have a file with channel locations specified in your folder in order to run all steps in the HAPPILEE pipeline. If channel locations are not provided, you will not be able to do the following: filter to channels of interest, detect and reject bad channels, interpolate bad channels, or re-reference your data. Each batch run of HAPPILEE must include files collected with the same channel layout (company, net type, and electrode number) and paradigm (resting-state or event-related), each of which users must specify for a given run. HAPPILEE processes data collected with any sampling rate, and files within a single run may differ in their individual sampling rates (if this is the case, we strongly recommend selecting the option to resample data to the same frequency to ensure subsequent steps perform comparably across files regardless of original sampling rate).

### 1.3 Line noise processing

HAPPILEE addresses electrical noise (e.g., 60 or 50 Hz artifact signal) through the multi-taper regression approach implemented by the CleanLineNoise program (Mullen, 2012). Multi-taper regression can detect and subtract regular sinusoidal signal at a given frequency (e.g. electrical noise) without sacrificing or distorting the underlying EEG signal at that frequency or nearby frequencies, drawbacks of the notch-filtering approach to line-noise processing (Mitra and Pesaran, 1999). Specifically, HAPPILEE applies the updated version of CleanLine's multi-taper regression (called CleanLineNoise, implemented in the PREP pipeline; Bigdely-Shamlo et al. 2015b) which is more effective in addressing line noise than the original CleanLine version present in HAPPE 1.0 (Gabard-Durnam et al., 2018a) software (purportedly a bug fix in the CleanLine code, see Makoto's pipeline page for unpublished evidence: https://sccn.ucsd.edu/wiki/Makoto%27s_preprocessing_pipeline#Why_does_IC_rejection_increase_gamma_power.2C_or_why_is_an_IC_not_broadband-independent). The legacy CleanLine version from HAPPE 1.0 (Gabard-Durnam et al., 2018) is available as an option to the user, however the updated version is registered as the default. CleanLineNoise's multi-taper regression scans for line-noise signal near the user-specified frequency ± 2 Hz, a 4-s window with a 1-s step size and a smoothing tau of 100 during the fast Fourier transform, and a significance threshold of *p* = 0.01 for sinusoid regression coefficients. This process is highly specific to the frequency of electrical noise, which the user can specify to be 60 Hz or 50 Hz. The user may also specify line-noise harmonic frequencies to be similarly cleaned (e.g. 30, 15 Hz, etc.) or neighboring frequencies to be cleaned (e.g. 59 and 61 Hz for a 60 Hz electrical signal). Quality control metrics for the degree of regular sinusoidal signal removal at line noise frequency/frequencies are automatically generated in HAPPILEE and discussed in detail as part of the subsequent “HAPPILEE Pipeline Quality Assessment Report” section of this manuscript.

### 1.4 Filtering

Filtering the EEG signal is important for isolating frequencies of interest and improving signal-to-noise (e.g., for ERP analyses or isolating frequencies within the range produced by EEG's electrophysiological sources) but can also distort the data in undesirable ways if attention is not paid to filtering settings both alone and in combination with other pre-processing steps (e.g., baseline correction, line-noise reduction steps; see Widmann et al. 2015 and Tanner et al. 2015 for thorough discussion of issues related to filtering EEG signals). For example, HAPPILEE uses CleanLine for line-noise removal rather than band-stop filtering (aka notch filtering) to avoid signal distortion (e.g., Luck 2005).

HAPPILEE uses the EEGLab filter pop_eegfiltnew (a zero-phase Hamming-windowed sinc FIR filter) to apply preliminary filtering prior to detecting bad channels (if user-selected) and employing artifact correction methods for all files. If processing resting-state EEG or task-based data for time-frequency analyses, the filtering at this stage is a band-pass filter from 1 to 100 Hz. Filtering at this stage of pre-processing allows the bad channel and artifact correction steps to assess the relevant frequencies for these types of analyses and optimizes performance in the higher frequency range. If the user selects the ERP option when entering user-inputs at the start of HAPPE 2.0, HAPPILEE applies a low-pass 100 Hz filter to aid the artifact correction steps that follow but does not apply any low-pass filtering at this stage. The liberal 100 Hz low-pass filter optimizes bad channel detection and artifact-correction performance with respect to EMG and other high-frequency artifact contamination in the data that occurs within but also beyond the higher frequencies typically-included in filtered ERPs. HAPPILEE then automatically interfaces with the HAPPE+ER pipeline for further filtering specific to ERPs after artifact correction (e.g., high-pass and low-pass filtering at user-specified values like 0.1 to 30 Hz, with options for filter type. See Monachino et al. 2021) for specifics on ERP filtering. Users may assess or explore the effects of different filter settings, i.e., filter type and frequency boundaries, on artifact-corrected data to optimize their ERP filtering using HAPPE's rerun functionality).

### 1.5 Bad channel detection (Optional)

HAPPILEE includes an option to detect channels that do not contribute usable brain data due to high impedances, damage to the electrodes, insufficient scalp contact, and excessive movement or electromyographic (EMG) artifact throughout the recording. Users have the option to run bad channel detection or not (in which case all channels are subjected to subsequent processing steps). Various methods are currently used to detect and remove bad channels across automated pipelines. However, some common automated detection methods used for high-density EEG may not be optimal for low-density EEG without modification, especially those relying heavily on standard deviation-related metrics of activity to detect outlier channels. For example, in HAPPE 1.0 (Gabard-Durnam et al., 2018), bad channel detection is achieved by evaluating the normed joint probability of the average log power from 1 to 125 Hz across the user-specified subset of included channels. Channels whose probability falls more than 3 standard deviations from the mean are removed as bad channels in two iterations of this bad channel detection step. However, removing channels that are three or more standard deviations from the mean activity assumes a normal distribution of channel activities (via the Central Limit Theorem) that we cannot assume with low-density channel numbers (Altman and Bland, 1995). Similarly, the FASTER algorithm (Nolan et al., 2010) used in the MADE pipeline (Debnath et al., 2020) flags channels by measuring each channel's Hurst exponent, correlation with other channels, and channel variance and standardizing the three values with an absolute Z-score (subject to the same constraints as standard deviations with very small samples). Thus, these algorithms require validation before implementation in low-density EEG data.

Other methods like EEGLab's Clean Rawdata algorithm (Kothe and Makeig 2013, additional code developed by Makoto Miyakoshi, Arnaud Delorme with Scott Makeig) may more readily translate to low-density EEG data. Specifically, Clean Rawdata's ‘Flatline Criterion,’ can detect channels with flat recording lengths longer than a user-specified threshold of seconds (indicating no data collected at that location). If the channel contains a flatline that lasts longer than the threshold, the channel is marked bad. Similarly, ‘Channel Correlation Criterion’ sets the minimally acceptable correlation value between the channel in question and all other channels. If a channel is correlated at less than the preset value to an estimate based on other channels, it is considered abnormal and marked bad. But features like the Line Noise Ratio Criterion, which identifies whether a channel has more line noise relative to neural signal than a predetermined value, in standard deviations based on the total channel population, should be assessed in the low-density EEG context.

To test the efficacy of FASTER (Nolan et al., 2010), HAPPE 1.0 (Gabard-Durnam et al., 2018), and Clean Raw data functions and determine the optimal criterion values for the detection of bad channels in low density data, we compared a series of thirty-three automated options to a set of manually identified bad channels for nineteen files in the BEIP dataset. For manual identification of bad channels, we took the ratings of three field experts and only selected files where agreement across reviewers was reached, ensuring that we were using clear-cut cases of good and bad channels within the optimization dataset. These files included channels that were bad for a variety of reasons and had variability in how many bad channels existed per file. For automated bad channel rejection, the files were run through the HAPPE 1.0 (Gabard-Durnam et al., 2018) legacy detection method for bad channels and the FASTER (Nolan et al., 2010) detection method used in the MADE pipeline (Debnath et al., 2020), as well as a number of iterations of the Clean Rawdata function and combinations of Clean Rawdata with spectrum evaluation to optimize channel classification (shown in Table 1). Note that for iterations of Clean Rawdata with Flatline Criterion included, the Flatline default of 5 s was determined to be sufficient for detecting flat channels and was not manipulated further. We evaluated the outputs from each criterion for bad channel detection relative to the manually selected channels by summing the number of false negatives and false positives for each file and calculating the overall accuracy rate across files for that set of automated parameters. False negatives refer to channels that were manually marked as bad but not flagged as bad by the pipeline. False positives refer to channels that were manually marked ‘good’ but were marked bad by the pipeline. An extra emphasis was placed on finding the settings with high accuracy that produced the lowest number of false positives in order to avoid getting rid of usable channels in the low-density dataset. HAPPILEE's optimal settings produced 12 false negative and 5 false positive channels across all 19 files (228 total channels), with an overall accuracy rate of 95.5%.

**Table 1:** Performance of bad channel detection parameters tested on nineteen files from the example dataset. Pre-wav denotes that spectrum evaluation was run prior to wavelet thresholding and post-wav denotes that spectrum evaluation was run following wavelet thresholding.

HAPPILEE combines EEGLab's Clean Rawdata functions with power spectral evaluation steps as follows. HAPPILEE first runs the Clean Rawdata ‘Flatline Criterion,’ to detect bad channels with flat recording lengths longer than 5 s (indicating no data collected at that location). After flat channels have been removed, HAPPILEE uses Clean Rawdata's ‘Line Noise Ratio Criterion’ with a threshold of 2.5 standard deviations (channels with line noise: neural data ratios greater than 2.5 standard deviations are marked as bad) and ‘Channel Correlation Criterion’ with a minimal acceptable correlation of 0.7 to detect additional bad channel cases. Finally, HAPPILEE includes a spectrum-based bad channel detection step following the Clean Rawdata functions. While the HAPPE 1.0 (Gabard-Durnam et al., 2018) method of legacy detection proved to be insufficient for our low-density dataset (see Table 1), evaluating the joint probability of average log power from 1 to 100 Hz was useful for optimizing bad channel detection alongside Clean Rawdata. A spectrum evaluation step with thresholds of −2.75 and 2.75 was included to optimize bad channel detection accuracy. Thus, HAPPILEE achieves bad channel detection that is suitable for low density data and expands the classes of bad channels that can be detected relative to HAPPE 1.0 (Gabard-Durnam et al., 2018) and MADE pipelines’ (Debnath et al., 2020) prior automated pipeline options.

### 1.6 Artifact correction in continuous data

Raw EEG data may contain a number of artifacts (e.g., from participant motion, electromyogenic activity, eye movements/blinks) that must be addressed during processing. Historically, artifact removal has been achieved via manual data inspection, where artifact-laden timepoints are deleted from the data, including artifact-free data from unaffected electrodes (i.e. artifact rejection approach). HAPPILEE instead uses wavelet thresholding methods for artifact correction (correcting artifacts without removing any timepoints) first to allow for fewer segments or trials to be rejected in subsequent artifact rejection steps and address artifacts that would survive segment rejection but could still impact the integrity of further analyses. This artifact correction approach is performed on each electrode independently in HAPPILEE, so it is appropriate for all channel densities down to single-electrode recordings, and its performance is channel-density independent (i.e., wavelet-thresholding will not perform differently in higher- vs. lower-electrode densities). These properties make wavelet-thresholding an excellent option for serving a variety of low-density EEG layouts.

Wavelet-thresholding refers to a series of three steps performed on each electrode:

Step 1) Apply the wavelet transform. Each electrode's time series is subjected to a wavelet transform by fitting a wavelet function to the EEG data to represent the signal and parse it into multiple frequency ranges (akin to frequency bands). Wavelet functions are orthonormal basis functions that come in a variety of shapes (called families) and are used for a variety of signal compression, denoising, and representation applications (including the authors’ personal favorite of detecting Van Gogh forgery paintings by fitting wavelet functions, Jafarpour et al., 2009). Wavelet functions used with electroencephalography resemble the oscillatory shape of EEG data (see Fig. 5 for examples in optimization section below). These wavelet functions also have excellent temporal resolution, so they can accurately represent both time and frequency information simultaneously. The wavelet transform passes the selected wavelet function over the EEG signal and produces a series of coefficients to describe how the wavelet function changed to fit the EEG signal's fluctuations across the entire timeseries. Importantly, poor function selection could result in poor fitting of the EEG signals, so the type of wavelet function used is important for the integrity of artifact-correction. The transform also separates the EEG signal into multiple frequency ranges and coefficients evaluate fluctuations in the signal within each frequency range separately. In this way, the wavelet transform operates like a frequency filter on the EEG signal, though one that is subsequently completely reversed and leaves no trace in the retained EEG data. The wavelet function has a resolution level (i.e., level of decomposition) associated with it that determines how many of these frequency ranges the EEG signal should be separated out into, with increasing resolution levels here parsing the lower frequencies into finer frequency bins. If the resolution level is not determined appropriately, unnecessary loss of low-frequency information can occur during thresholding (described below). Once the wavelet transform separates out and fits the EEG signal in these frequency bins in Step 1, artifacts may be detected in Step 2.
Step 2) Threshold the data to isolate artifact signal. The wavelet transform coefficients that describe the EEG signal within each frequency bin are subjected to a thresholding procedure to separate out artifact signal from neural signal. This feature allows for frequency-band-specific artifact detection relative to neural data in those same frequencies, which is a key component of wavelet-thresholding's success in EEG signals. Given that artifact signal is larger than the neural signal found at the same frequencies across all classes of artifact, and occurs more inconsistently throughout the timeseries than neural-related fluctuations, the wavelet coefficients reflecting these properties are separated out as artifact-signal to be removed from the data using a thresholding method and rule. If the wavelet resolution level is not set appropriately, low-frequency neural data, which typically have higher amplitudes than higher-frequency data in EEG signals, can be mistakenly removed as artifacts. There are multiple methods for determining the threshold for determining signal to be artifact vs. neural. HAPPILEE uses an empirical Bayesian method that learns from each individual's EEG where to set the threshold and is less sensitive to outlier effects (Clyde and George, 2000). This threshold may be determined and applied across all frequency ranges (level-independent threshold) or determined individually for each frequency range based on the characteristics of the signal in that frequency range (level-dependent threshold). HAPPILEE uses a level-dependent threshold to best fit the artifact properties occurring within each frequency range. Once the threshold has been applied to determine which wavelet coefficients (reflecting parts of the EEG signal) are above the threshold, they can be removed as artifact-related signal. There are also multiple methods for treating these thresholded coefficients in terms of how they are separated from the rest of the signal called threshold rules, but HAPPILEE uses a hard threshold that completely removes the sub-threshold coefficients from the data (i.e., completely separates the signal classified as neural from the artifact-related signal). Other rules like the soft threshold instead downweight the sub-threshold coefficients closest to the threshold cutoff in the data, for example. If these steps together are not optimized for EEG signals, incomplete artifact correction, or attrition of neural signal can both occur, so great care was taken in optimizing these parameters for HAPPILEE (described below). Once the artifact-signal is fully separated from the neural signal, it can be removed from the electrode timeseries without disturbing the underlying neural signal at those timepoints via the inverse wavelet transform and subtraction.
Step 3) Apply the inverse wavelet transform and subtract thresholded (artifact) signal. Finally, the artifact-related coefficients are transformed back from wavelet-coefficients to the electrode's signal timeseries using the inverse of the wavelet transform function. The HAPPILEE wavelet function family allows for this transform without distortion to the data in phase, amplitude, or frequency space. This inverse transform thus results in an artifact-timeseries that is then simply subtracted from the electrode's original timeseries, resulting in an artifact-corrected timeseries. Because waveleting is both a time- and frequency-specific method, the artifact timeseries will be 0 where no artifact is temporally present in the data, and thus this subtraction does not disturb the clean EEG signal surrounding the artifact-contaminated segment at all (this distinguishes wavelet-thresholding from other artifact-correction strategies like independent component analysis that in practice do not consistently meet this standard in the artifact-labeled components). The wavelet-thresholded artifact-corrected signal may then continue through pre-processing steps like segmentation.

Through only three steps, the wavelet-thresholding process contains many parameters that may be optimized to improve artifact-correction performance. The final wavelet-thresholding parameters implemented in HAPPILEE are as follows: ‘Wavelet Family,’ ‘coif4;’ ‘Level of Decomposition,’ ‘10;’ ‘Noise Estimate,’ ‘Level Dependent;’ ‘Thresholding Method,’ ‘Bayes;’ ‘Threshold Rule,’ ‘Hard’. The approaches and steps to optimize wavelet-thresholding artifact correction are detailed below, with additional details about each component of the wavelet-thresholding process.

#### 1.6.1 Wavelet threshold optimization approaches

Three approaches were taken to test and optimize automated wavelet thresholding-based artifact correction in HAPPILEE using the BEIP dataset. Prior to artifact correction testing, all files were initially filtered and subjected to line noise correction. The approaches are detailed below.

**Fig. 3:**
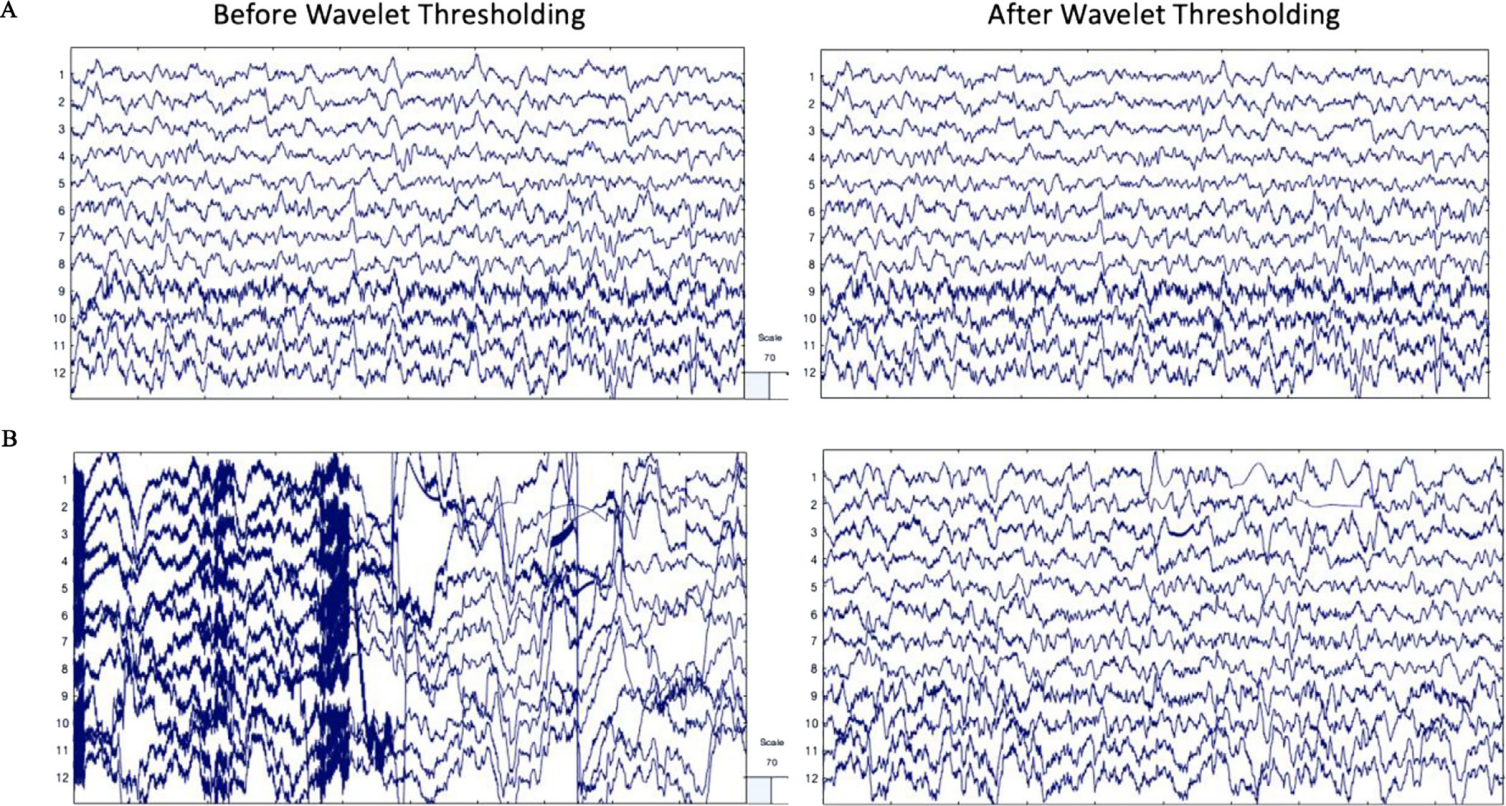
EEG signal before and after wavelet thresholding with the following parameters that were optimized for low density data: ‘Wavelet Family,’ ‘coif4;’ ‘Level of Decomposition,’ ‘10;’ ‘Noise Estimate,’ ‘Level Dependent;’ ‘Thresholding Method,’ ‘Bayes;’ ‘Threshold Rule,’ ‘Hard.’ Two files from the same participant in the example dataset are shown with 10 s of data extracted from the clean 30 s segment (A) and artifact-laden 30 s segment (B). The EEG signal before processing is shown in the left panel. The EEG signal after wavelet thresholding is shown in the right panel. All scales are in microvolts.

The first approach (the clean vs. artifact approach) involved selecting two 30 s segments within each participant's EEG file. The first 30 s segment was heavily artifact laden while the second 30 s segment was determined to be clean and without considerable artifact by experienced researchers. This approach facilitated testing whether artifact was effectively and accurately removed via wavelet thresholding. That is, optimal artifact correction performance would be characterized by (1) substantial (artifact) signal removal for the artifact-laden 30 s file, indicating sensitivity to artifacts, and (2) minimal signal removal for the clean 30 s file from the same individual, indicating specificity in signal removal constrained to artifact. High levels of signal removal in the clean 30 s files would indicate unnecessary data loss, while low levels of signal removal in the artifact-laden 30 s files could indicate insufficient performance. Additionally, to ensure that artifact removal was not biased to certain frequencies, data correlations pre- and post-processing were evaluated at key frequencies spanning all canonical frequency bands in the clean and artifact-laden segments. Examples of the clean and artifact signals within an individual (before and after wavelet thresholding) are provided in Fig. 3.

**Fig. 4:**
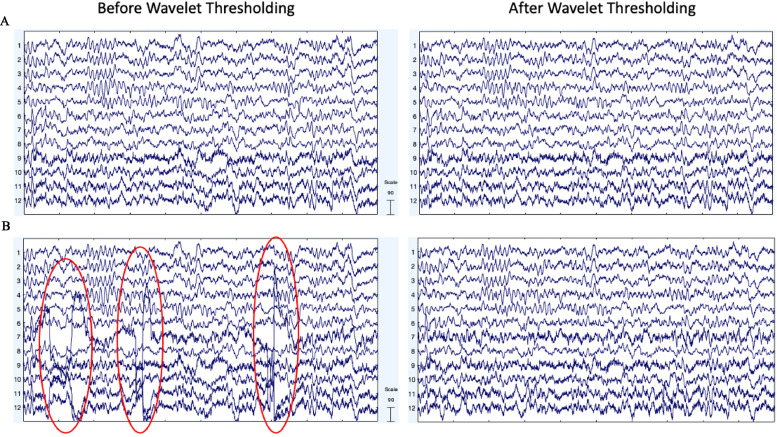
EEG signal before and after wavelet thresholding with the following parameters that were optimized for low density data: ‘Wavelet Family,’ ‘coif4;’ ‘Level of Decomposition,’ ‘10;’ ‘Noise Estimate,’ ‘Level Dependent;’ ‘Thresholding Method,’ ‘Bayes;’ ‘Threshold Rule,’ ‘Hard.’ Two files from the same participant in the example dataset are shown with 10 s of data extracted from the clean 30 s segment (A) and artifact-added 30 s segment (B). The EEG signal before processing is shown in the left panel. The EEG signal after wavelet thresholding is shown in the right panel. All scales are in microvolts.

The second approach (artifact-addition approach) used known artifact signals to establish how much artifact could be removed without distorting underlying neural signals during wavelet thresholding. In the absence of a ground-truth neural signal, we used the 30 s clean files from the first approach as the signal to be recovered during artifact-correction. We then isolated artifact timeseries by running ICA on the artifact laden 30 s files and selecting approximately 2 components per individual that were determined to be artifact with minimal neural data via visual inspection and automated classification through both the ICLabel and Multiple Artifact Rejection Algorithm options. We subsequently added those artifact timeseries to the clean 30 s data segment from the same individual (see Supplemental File 1 for types of artifacts added for each file). These 30 s files with added artifact were then subjected to wavelet-thresholding and compared (via correlation coefficients) to the clean 30 s files run through wavelet-thresholding to determine how much of the added artifact was removed during the artifact-correction step (example from single participant in Fig. 4). Higher correlation coefficients would indicate better recovery of the clean EEG signal and better removal of the added artifact during the wavelet-thresholding step. (The post-wavelet thresholded clean files were used as the comparison because even these clean files had some minor artifacts that could be removed during artifact correction, so the post-wavelet-thresholded versions were the cleanest option for comparisons).

For the third approach, we used simulated EEG data with artifact added to it in order to have a “ground truth” signal that we could attempt to recover with waveleting. To create the simulated EEG data, we used code from Bridwell et al. (2018). In short, the simulated EEG consisted of four signals. The four signals had distinct spatial patterns and frequency ranges (1.00–3.91, 3.91–7.81, 7.81–15.62, and 15.62–31.25 Hz). For a more thorough description on how the simulated signals were created, (see Bridwell et al. 2018). After creating the simulated EEG data, we added various developmental artifacts and adult eye blinks to the data to test multiple waveleting settings and wavelet thresholding in general. To get the blink artifact, we used a clear blink independent component (IC) from an adult participant (see Leach et al. 2020 for the specific study details). This IC was selected based on both an automated artifactual IC detection algorithm and visual inspection by two researchers with over five years of EEG and at least two years of ICA experience. For the developmental artifact, we pulled eight ICs from the BEIP dataset used above in the artifact addition approach. After adding the artifact to the simulated EEG data, we did a 1 Hz highpass and 35 Hz lowpass filter. Following this, we epoched the data into two-second epochs (50% overlap) to prepare the data for wavelet thresholding and/or artifact rejection. For artifact rejection, we used a −100 to 100 μV voltage threshold to identify bad epochs. We also required both frontal electrodes to exceed this threshold in order to classify an epoch as containing a blink. In order to test how well wavelet thresholding removes artifact without removing neural activity, we computed the power spectral density (PSD) on the original simulated signal (no artifact added) and compared that to the PSD of the signal after pre-processing with various wavelet thresholding parameters when either one or two developmental artifactual independent components and an adult eye blink component are added to the simulated signal. In addition, we looked at how the preprocessed data might differ when only artifact rejection is run compared to when waveleting is run before artifact rejection. This gave us two pre-processing conditions: (1) Artifact rejection only (no wavelet thresholding) and (2) Wavelet thresholding followed by artifact rejection.

These three distinct approaches facilitated optimizing and evaluating the wavelet thresholding method for artifact removal in low-density data in multiple ways. Importantly, the wavelet thresholding approach broadly includes decomposing the EEG signal via a wavelet transform, determining a threshold value or values used to dissect data into the portion to be retained and the portion to be rejected, removal of the rejected data components, and reconstruction of the remaining signal. Each of these steps may be accomplished multiple ways across wavelet thresholding methods. Here, five key parameters in the wavelet thresholding process were manipulated and tested to optimize wavelet thresholding performance in this context, specifically: wavelet family, wavelet resolution (i.e., level of data decomposition), noise estimate method, thresholding level, and threshold rule. Each parameter was manipulated one at a time within a default set of wavelet thresholding parameters and tested using the clean vs. artifact approach and artifact-added approach in the BEIP dataset. The initial default set of wavelet thresholding parameters was chosen based on preliminary visual inspection of performance across a broader range of parameters prior to optimization and was as follows: ‘Wavelet Family,’ ‘coif5;’ ‘Level of Decomposition,’ ‘8;’ ‘Noise Estimate,’ ‘Level Dependent;’ ‘Thresholding Method,’ ‘Bayes;’ ‘Threshold Rule,’ ‘Soft.’ Subsequent optimization of each parameter is described in detail below.

#### 1.6.2 Wavelet family

**Fig. 5:**
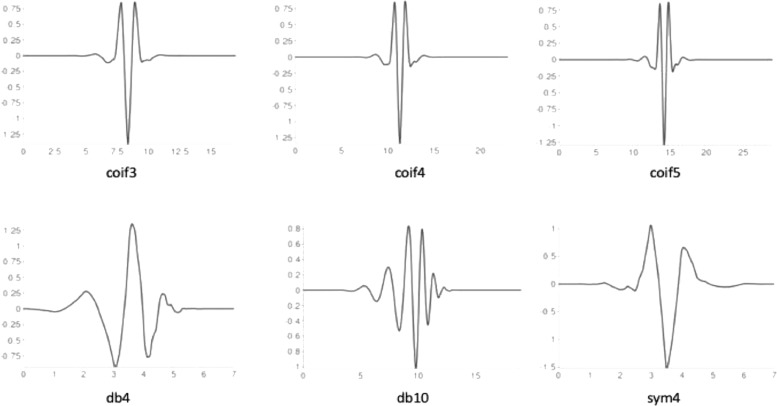
Visualization of the three wavelet families with selected orders tested to find the optimal wavelet function to carry out stationary wavelet transform on low density data.

The wavelet-thresholding method first subjects each electrode's time series to wavelet transform by fitting a wavelet function to the data. The wavelet transform produces a series of coefficients to describe the EEG signal's fluctuations across multiple frequency ranges. The wavelet function consists of both a wavelet family, dictated by the mother wavelet shape (e.g., the Coiflet mother wavelet is more symmetric than the Daubechies mother wavelet), and the wavelet order, which modifies the mother wavelet shape (see Fig. 5; i.e., Coiflet order 4 wavelet has 8 vanishing moments in the function). To find the optimal wavelet function to carry out stationary wavelet transform, we tested the Coiflets family (orders 3, 4, and 5), the Daubechies family (orders 4 and 10), and the Symlets family (order 4). We selected these family/order combinations as they share shapes similar to those found in EEG signals and they are all orthogonal wavelet functions, which optimizes decomposition and reconstruction of the EEG signal from the wavelet transform (Strang and Nguyen, 1996). Moreover, prior literature indicates these wavelet families and specific orders have performed well on electrophysiological data (Al-Qazzaz et al., 2015; Alyasseri et al., 2017; Harender and Sharma, 2018; Lema-Condo et al., 2017; Nagabushanam et al., 2020). Using these wavelet families, we evaluated whether there was biased data removal at any of the data frequencies in the clean 30 s segments by evaluating correlations between data pre-waveleting and post-waveleting at specific canonical frequencies. We also compared data removal rates between the clean files and the 30 s artifact laden files. As a second analysis, we evaluated which wavelet family and order combination removed the most added artifact across frequencies (i.e., which wavelet facilitated the greatest correlation between the artifact added files and the clean files post-processing).

After running the various wavelet family/order options, we found there were not meaningful differences between several wavelet family/order options (Tables 2 and 3). Specifically, performance did not differ between coif4, coif5, db4, and sym4 options across the clean vs. artifact-laden and artifact addition tests (e.g., correlation values in the artifact-addition tests were identical to the hundredths place). Coif4 was selected as the wavelet implemented in HAPPILEE as this wavelet/order also performed very well in data collected from a saline-based system (EGI), suggesting its performance may generalize more broadly than the other options tested here (Monachino et al., 2021).

**Table 2:** **Artifact-laden vs. Clean Approach.** Correlations between the EEG signal before wavelet thresholding and the EEG signal after wavelet thresholding for the wavelet parameters tested on the artifact-laden and clean 30 s segments. The *r* values of the wavelet parameters that are included in the final code are bolded. Asterisks denote default wavelet parameters.

**Table 3:** **Artifact-added approach**. Correlations between the EEG signal before wavelet thresholding and the EEG signal after wavelet thresholding for the wavelet parameters tested on the artifact-added 30 s segments. The *r* values of the wavelet parameters that are included in the final code are bolded. Asterisks denote default wavelet parameters.

#### 1.6.3 Wavelet resolution/level of data decomposition

Following wavelet family/order selection, we manipulated the resolution of the wavelet that affects the level of data decomposition in wavelet thresholding. Specifically, this level of decomposition determines how fine-grained the frequency bands are in which data correction occurs. Importantly, in the current code, the data sampling rate (not for example, the frequencies retained through initial filtering) determines which frequencies fall into different levels of decomposition. For example, the first level of decomposition for a file sampled at 1000 Hz (regardless of frequency filtering) would split data into two halves around 500 Hz. If that file had been resampled to 500 Hz prior to wavelet decomposition, the first level of decomposition would now split data into halves around 250 Hz. The default decomposition level splits data down to ≤1 Hz. We tested decomposition levels of ∼4, 2, and 1. The 4 Hz decomposition resulted in increased data removal in the lower frequencies relative to other frequencies of the clean data, resulting in data correlations pre-/post-thresholding of less than 0.5 (e.g., *r* = 0.48 at 2 Hz). This pattern indicated the need for further decomposition levels to avoid over-rejecting low frequency data that is not artifact-laden (low frequencies were rejected at similar rates in the artifact-laden data). In the clean vs. artifact files, we saw no difference in which data was rejected and retained when comparing levels 2 and 1 Hz, though 1 Hz provides coverage down to the filtering cutoff for time-frequency analyses. Therefore, HAPPILEE decomposes data into detail coefficients for frequencies above approximately 1 Hz to evaluate artifacts within each frequency range for time-frequency-related analyses. For event-related-potential decomposition optimization where signals below 1 Hz are relevant, HAPPILEE interfaces with the HAPPE+ER pipeline and uses those settings, so we refer readers to the HAPPE+ER manuscript (Monachino et al., 2021).

#### 1.6.4 Noise (Artifact) estimation level

Once the level of decomposition is set, the noise estimate parameter is chosen to establish either a threshold for each level of decomposition (level dependent threshold) or establish a threshold that operates across all of the levels of decomposition (level independent threshold). The threshold(s) determine which wavelet coefficients describe data that is artifact-laden (i.e., coefficients describing larger amplitude changes, or noise) and will be removed from the EEG data during this artifact correction step. Due to the unavailability of level dependent thresholding for the wavelet function used at the time of its conception, HAPPE 1.0 (Gabard-Durnam et al., 2018) employed level independent thresholding (with a different threshold method and rule as well). We anticipated that level dependent thresholding would improve artifact detection specificity because of its ability to scale within each frequency range (e.g., artifacts in gamma frequencies have smaller amplitudes than artifacts in delta frequencies), rather than apply across all frequencies at once (which may over-penalize low-frequency clean EEG data that has higher amplitudes than higher-frequency clean EEG data). The default set of parameters was run with both level dependent and level independent thresholding and confirmed our prediction. With the improved thresholding method and rule included in the default parameters, the level independent threshold now heavily over-rejected the clean data (resulting in a correlation pre-/post-thresholding of *r* = 0.02), removing nearly all of the data (and a similar level of data removal was observed in the artifact-laden data). (Note: this performance differs from HAPPE 1.0 (Gabard-Durnam et al., 2018) due to the other wavelet thresholding parameter changes included in the new default settings of HAPPILEE, and does not reflect the functionality of wavelet-independent thresholding in HAPPE 1.0). There was no meaningful difference between level independent and level dependent thresholding in how much artifact was removed in the artifact-added approach. This pattern of results suggested the level dependent threshold was just as effective at removing artifact as the level independent threshold without also removing underlying clean neural signal. As a result, a level dependent threshold was chosen in order to preserve data without compromising artifact correction success.

#### 1.6.5 Thresholding method and threshold rule

**Fig. 6:**
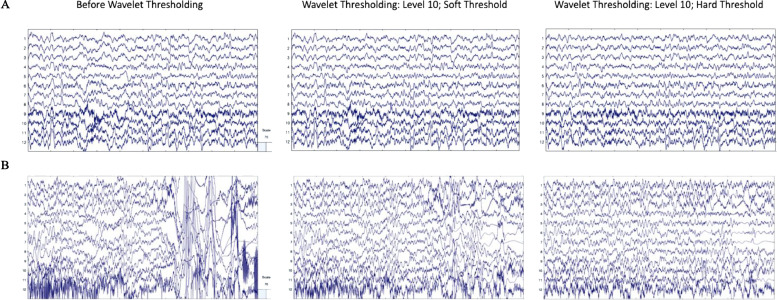
EEG signal before and after wavelet thresholding with the following parameters: ‘Wavelet Family,’ ‘coif5;’ ‘Level of Decomposition,’ ‘10;’ ‘Noise Estimate,’ ‘Level Dependent;’ ‘Thresholding Method,’ ‘Bayes;’ ‘Threshold Rule,’ ‘Soft’ (middle panel) and ‘Wavelet Family,’ ‘coif5;’ ‘Level of Decomposition,’ ‘10;’ ‘Noise Estimate,’ ‘Level Dependent;’ ‘Thresholding Method,’ ‘Bayes;’ ‘Threshold Rule,’ ‘Hard’ (right panel). Two files from the example dataset are shown with 10 s of data extracted from the clean 30 s segment (A) and artifact-laden 30 s segment (B). The EEG signal before processing is shown in the left panel. The EEG signal after wavelet thresholding with a soft threshold is shown in the middle panel. The EEG signal after wavelet thresholding with a hard threshold is shown in the right panel. All scales are in microvolts.

Wavelet coefficients are then subjected to thresholding, (here, in a level-dependent way) such that coefficients with values smaller than a determined threshold for that level have their contribution to the data substantially suppressed (similar to Jansen, 2001; You and Chen, 2005). For EEG data, this effectively isolates the artifact signals within each frequency level (which are then subtracted out of the original EEG signal to clean it). A number of high performing options to determine the thresholds separating artifact from clean EEG have been established in the literature (Anumala and Kumar Pullakura, 2018; Estrada et al., 2011; Geetha and Geethalakshmi, 2011a; Geetha and Geethalakshmi, 2011b; Guo et al., 2020; Jiang et al., 2007), specifically ‘Empirical Bayes’ (Johnstone and Silverman, 2004), ‘SURE’ (Donoho and Johnstone, 1995), ‘Universal’ (Donoho, 1995), and ‘Minimax’ (Donoho and Johnstone, 1998) thresholding approaches. HAPPE 1.0 (Gabard-Durnam et al., 2018) originally included a Universal Threshold approach, but we aimed to examine these additional high-performing options as well to find the best fit for the current version of the pipeline. Relatedly, we evaluated available threshold rules for the various thresholding methods, specifically ‘Soft’ (Guo et al., 2002; Donoho, 1995), ‘Median’ (Abramovich et al., 1998; Clyde and George, 2000; Johnstone and Silverman, 2005), and ‘Hard’ (Guo et al., 2002). Of note, not all thresholding methods are compatible with all thresholding rules. For example, the median rule is specific to the thresholding method ‘Bayes’ as it involves using the median posterior generated by the Bayesian algorithm to determine the threshold. Soft and hard threshold rules are different in how they treat the coefficients near the threshold (in soft thresholding, these coefficients are shrunk while they are unaffected in hard thresholding). For thresholding options, we found minimal difference between options when tested on the artifact-added data, but we were able to eliminate ‘SURE’ due to over-rejecting data in the clean files (resulting in a correlation pre-/post-thresholding of *r* = 0.77). ‘Bayes’ narrowly outperformed ‘Minimax’ on the artifact-added data (Table 3), but the reverse was true for our clean and artifact-laden data (Table 2). ‘Bayes’ was chosen as the thresholding method as it considers the uncertainty of the potential artifact and has been shown to result in more accurate denoising of signals generally. Moreover, the Bayes algorithm increases performance with increased data samples (as it can adjust its certainty estimates about artifact from more data), so performance on these 30 s files is a conservative reflection of this general performance. For threshold rule, on average, the median and hard thresholds removed more of the clean and artifact-laden file data, especially at the lower frequencies (e.g., at 2 Hz in clean data: soft threshold *r* = 0.84, median threshold *r* = 0.79, hard threshold *r* = 0.63, see Table 2). However, visual inspection of data cleaned using soft vs. hard thresholds revealed that hard thresholds appeared to better remove artifact (see Fig. 6). Therefore, this step was further explored using the simulated signals as described below and ultimately a Bayesian method with hard thresholding rule was implemented.

**Fig. 7:**
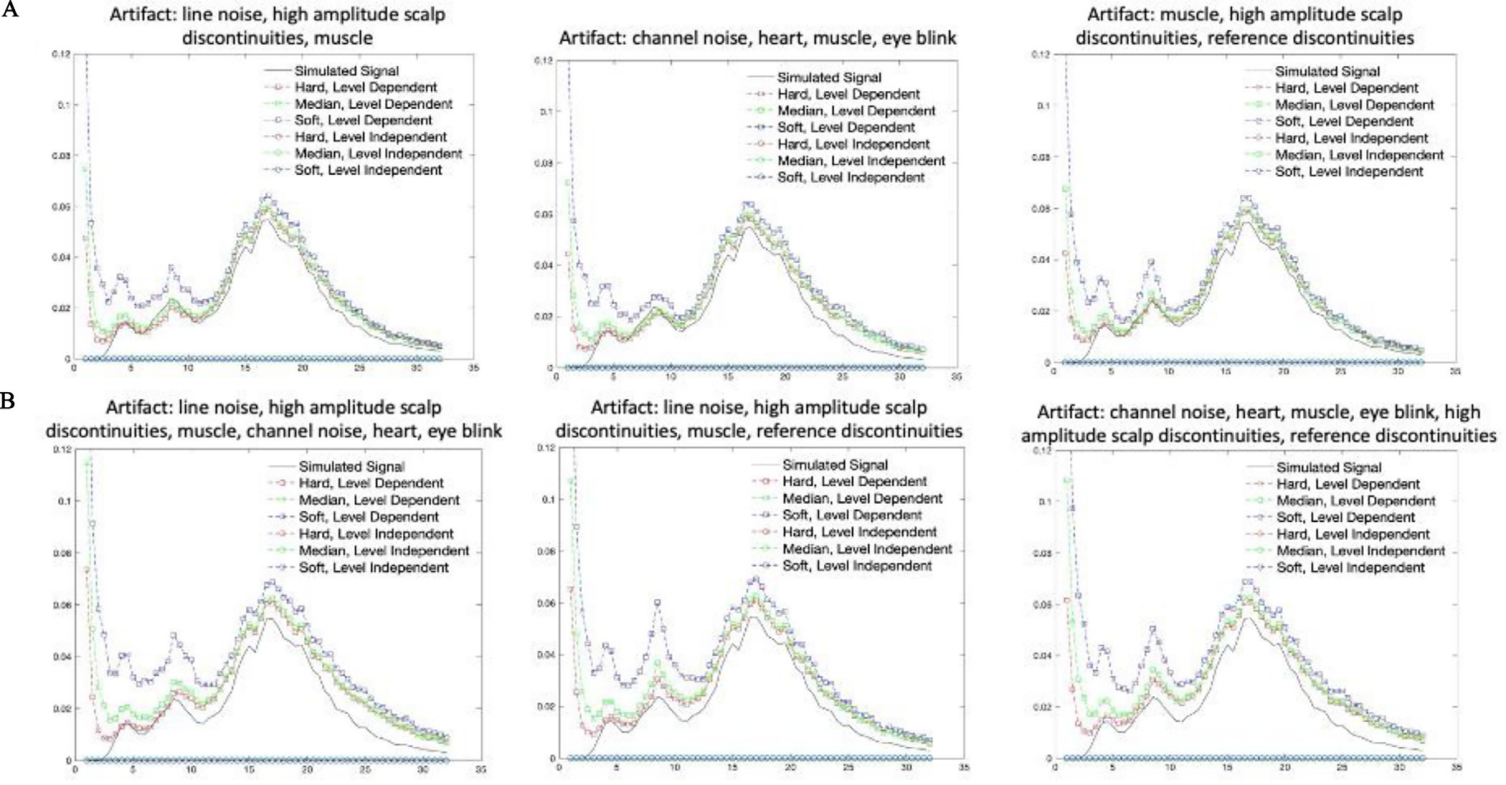
Plots of the power spectral density for the simulated signal without artifact and the simulated signal with an adult eye blink component added and either one developmental artifactual IC added (A; top panel) or two developmental artifactual ICs added (B; bottom panel) following wavelet thresholding.

For the third approach with simulated data that had real artifact added to it, we tested which combination of noise estimation level and threshold rule performed best in terms of removing artifact while retaining the ground-truth underlying simulated signal. We compared combinations of level dependent and level independent estimation methods with soft, median, and hard threshold rules (Fig. 7). We tested on the simulated signal with an adult eye blink component added and either one developmental artifactual IC added or a combination of two developmental artifactual ICs added that reflected a variety of different artifact types and combinations to ensure generalization of the results across artifact conditions (The specific ICs added to the simulated signal are provided above each plot in Fig. 7). We found that level dependent thresholding far outperformed level independent thresholding. This is not surprising given that level dependent thresholding scales within each frequency range (and thus may be more sensitive to different artifact profiles across frequencies) rather than evaluating all frequencies together as is the case for level independent thresholding (see Fig. 7, level-independent results all lie along the x-axis). Moreover, level dependent thresholding with a hard or median threshold rule outperformed level dependent thresholding with a soft threshold rule in terms of visually returning the simulated signal's spectrum profile. The hard threshold rule narrowly outperformed the median threshold rule (especially for lower frequencies). Together these results helped solidify the decision to use level dependent thresholding with a hard threshold rule as the combination of parameters that best removed artifact while retaining the ground-truth underlying signal.

Taken together, the final wavelet-thresholding optimized parameters implemented in HAPPILEE are again as follows: ‘Wavelet Family,’ ‘coif4;’ ‘Level of Decomposition,’ ‘10;’ ‘Noise Estimate,’ ‘Level Dependent;’ ‘Thresholding Method,’ ‘Bayes;’ ‘Threshold Rule,’ ‘Hard’. These parameters ensure optimized wavelet-thresholding based artifact correction occurs in EEG data.

### 1.7 Segmentation (Optional)

After artifact correction, HAPPILEE includes an optional data segmentation step along with several additional artifact rejection steps to further optimize processing. For data without event markers (e.g., resting-state EEG), regularly marked segments of any duration specified by the user are generated for the duration of the recording (e.g., 1 s segments). For low density data with event markers (e.g., event-related EEG data or ERP designs), data can be segmented around events as specified by user inputs (ERP-processing is supported, including baseline and timing offset correction; see Monachino et al., 2021 for more information).

Users with data files where segment rejection would lead to an unacceptably low remaining number of segments for analysis may choose an optional post-segmentation step involving the interpolation of data within individual segments for channels determined to be artifact-contaminated during that segment, as implemented by FASTER software (Nolan et al., 2010). Each channel in each segment is evaluated on the four FASTER criteria (variance, median gradient, amplitude range, and deviation from mean amplitude; Nolan et al. 2010), and the Z score (a measure of standard deviation from the mean) for each channel in that segment is generated for each of the four metrics. Any channels with one or more Z scores that are greater than 3 standard deviations from the mean for an individual segment are marked bad for that segment. These criteria may identify segments with residual artifacts. Subsequently, for each segment, the channels flagged as bad in that segment have their data interpolated with spherical splines, as in FASTER (Nolan et al., 2010). This allows users to maintain the maximum number of available segments, while still maximizing artifact rejection within individual segments. However, we caution users from implementing this option in cases where channels are distributed with significant distance between them as the interpolation process would pull data from distal channels that does not reflect the appropriate activity profile for that scalp space. Effects of interpolation on data may depend on experiment, layout (Melnik et al., 2017; Bigdely-Shamlo et al., 2015a), and interpolation method (Courellis et al., 2016; Petrichella et al., 2016; Robeson, 1997).

The majority of users, including those who wish to avoid interpolating data within individual segments, may instead choose to reject segments that are determined to still be artifact-contaminated. HAPPILEE includes three segment rejection options. Criteria for rejection include a choice of joint-probability criteria, amplitude-based criteria, or a combination of joint-probability criteria with amplitude-based criteria. Joint-probability criteria considers how likely a segment's activity is given the activity of other segments for that same channel, as well as other channels’ activity for the same segment. The assumption is that artifact segments should be the rare segments with activity several standard deviations apart relative to the rest of the data. Amplitude-based criteria sets a minimum and maximum signal amplitude as the artifact threshold, with segments being removed when their amplitude falls on either side of this threshold. HAPPILEE allows the user to specify their minimum and maximum allowable amplitudes. Users may also specify a combined approach with joint-probability criteria and amplitude-based criteria that removes outlier segments that fail either standard deviations or the signal amplitude criteria.

To test the efficacy of the three segment rejection options and determine the optimal criterion values for the rejection of segments in low density EEG data, we compared a series of ten automated options to a set of manually rejected segments for fourteen files in the BEIP dataset. We manipulated the standard deviation values for joint-probability rejection, the amplitude values for amplitude-based rejection, and tested combinations of different joint-probability standard deviations with amplitude criteria. The number of segments rejected for each of these automated rejection approaches was compared to the segments rejected via manual inspection as the gold standard approach using paired t-tests (Table 4). Amplitude-based rejection alone did not sufficiently match the manual rejection rates. However, the number of segments rejected using joint-probability criteria of 2 standard deviations alone or in combination with amplitude criteria (here, −150 and 150 microvolts) were not significantly different from the number of segments rejected manually (both *p* > 0.1).

**Table 4:** Comparison of various segment rejection options tested on nineteen files from the example dataset. *P* values are calculated from t-tests comparing the number of remaining segments for each parameter following automated rejection to the number of segments remaining following manual rejection.

Segment rejection performance was further evaluated for these two approaches by comparing the identity of segments rejected via the automated approach to the manually rejected segments by summing the number of false negatives and false positives for each file and calculating the overall accuracy rate across files compared to the manual rejection classification (i.e., did HAPPILEE reject the same segments that were rejected manually). The joint probability criterion alone (using 2 standard deviations) achieved the higher accuracy rate of 91.2% across all files but joint-probability with amplitude also did well (91.0% accuracy) (see Table 5). HAPPILEE therefore includes three segment rejection options with the following recommendations. For data with sufficient channels (e.g., here 12 was sufficient but we did not test performance on sparser configurations), segment rejection via joint-probability criteria is recommended. This setting is also recommended for users combining data collected across different systems or ages where the overall signal amplitude may differ across files. Users performing analyses in the time domain (as for ERP paradigms) may opt to include amplitude-based criteria. For users with very low-density configurations where the joint-probability criteria relying on standard deviations may not perform as well as it did here, amplitude-only criteria may be used for segment rejection.

**Table 5:** Performance of segment rejection parameters tested on nineteen files from the example dataset.

### 1.8 Interpolation of bad channels (if bad channel detection was run)

For all HAPPILEE runs that included bad channel detection, channels marked as bad are now subject to spherical interpolation (with Legendre polynomials up to the 7th order) to repopulate their signal. The identity of all interpolated channels, if any, for a file are recorded in the HAPPILEE processing report for users who wish to monitor the percentage or identity of interpolated channels in their datasets before further analysis. This interpolation step is available for all files formats except for .mat formats without channel locations as the interpolation step requires electrode location information to interpolate appropriately spatially. However, similar to segment interpolation outlined above, we caution users against including these interpolated channels in analyses in cases where channels are distributed with significant distance between them as the interpolation process would pull data from distal channels that does not reflect the appropriate activity profile for that scalp space. Effects of interpolation on data may depend on experiment, layout (Melnik et al., 2017; Bigdely-Shamlo et al., 2015a), and interpolation method (Courellis et al., 2016; Petrichella et al., 2016; Robeson, 1997). Because this step is not optional when bad channel detection is run, if a user wishes to omit these interpolated channels from analyses, a list of interpolated channels can be found on the data quality assessment report.

### 1.9 Re-referencing (Optional)

HAPPILEE offers users the choice to re-reference the EEG data if they wish. While there is no ideal reference for EEG, re-referencing with one of several practical if imperfect options can reduce artifact signals that exist consistently across electrodes, including residual line-noise, and recover signal from online reference channels of interest. If re-referencing, the user may specify either re-referencing using an average across all channels (i.e., average re-reference), using a channel subset of one or multiple channels, or re-referencing to a point at infinity using the reference electrode standardization technique (REST) (for additional information on REST, see Yao 2001). It is important to carefully consider which re-referencing method is best suited for a particular dataset, especially when using a low-density layout (e.g. Junghofer et al. 1999). Users may re-process data with different re-referencing options selected to assess the effect of re-reference scheme on their results.

A major concern that stems from low density layouts is inter-electrode distance. Given the limited number of channels, there may be cases where the electrodes used are far apart spatially on the scalp, leading to biases when re-referencing to a channel subset of one or multiple channels. If the reference electrode is spatially close to some electrodes, but far from others, then it will not be representative of the signal as a whole across the scalp and be disproportionately influenced by the immediately surrounding electrodes (Lei and Liao, 2017). If the user chooses to re-reference to a channel subset, they must ensure that the amplitude of that subset is representative of the broader signal across other electrode sites and that the signal at the chosen electrode(s) is not correlated with task-induced activity (Kim, 2018).

To avoid biases associated with re-referencing to electrodes on the scalp, average re-referencing is often used, averaging across all scalp electrodes. However, this can still be challenging with low density datasets when the number of electrodes are limited and the distribution of the electrodes are uneven across the scalp (Dien, 1998). Average re-referencing is recommended when the EEG layout is dense (some recommend over 100 channels) and evenly distributed, allowing the overall activity to average to 0 (Peng and Peng, 2019). Users should consider the distribution of their electrodes before using average re-referencing on low density data.

REST, proposed as a neutral reference to a point at infinity (Yao, 2001), is another option for low density data. In a study considering re-referencing for layouts with 32, 64, and 128 channels, Lei and Liao (2017) found that the relative error was lowest for REST, followed by average re-reference, and then referencing to a single electrode (FCz, Oz), regardless of the number of electrodes and signal-to-noise ratio. An additional study looking at a graph-based analysis of brain connectivity using a 19-channel layout found that REST can minimize contamination and reduce effects of volume conduction (Olejarczyk and Jernajczyk, 2017). With an even broader range of electrodes (21, 34, 74, 128 electrodes), Chella et al. (2016) found that REST reduces biases associated with referencing to a singular electrode and average re-referencing across all electrode density layouts, although REST's performance was further improved with high-density layouts. REST has been implemented in HAPPILEE using the open-source MATLAB toolbox for REST of scalp EEG (Dong et al., 2017).

Note that for re-referencing to a subset of channels and average re-referencing, only channels within the user-specified channel subset selected for HAPPILEE processing can be used for re-referencing. During re-referencing, if there is a prior reference channel (e.g., an online reference channel), that channel's data is recovered and included in the re-referenced dataset. For example, EGI (Electrical Geodesics, Inc., Eugene, OR) data is typically online-referenced to channel CZ. In this example, users could now recover data at channel CZ by re-referencing to any other channel or channels (or average rereference) in this step.

**Fig. 8:**
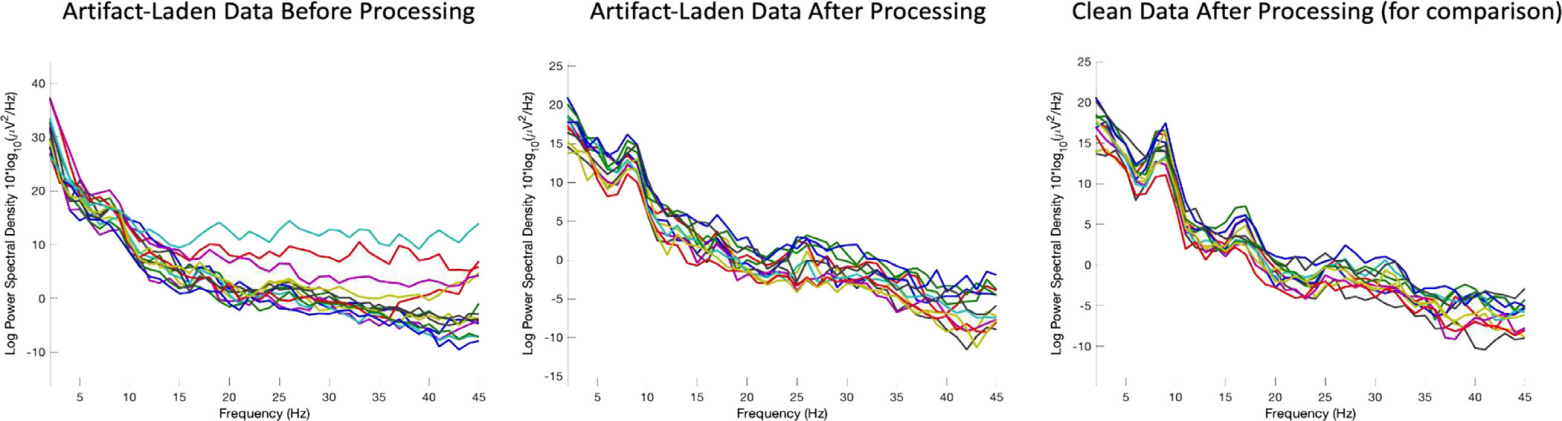
EEG spectrum before and after complete processing through HAPPILEE with the final optimizations for each step. Two files from the same participant in the example dataset are shown from the artifact-laden 30 s segment and clean 30 s segment. The artifact-laden EEG spectrum before processing is shown in the left panel. The artifact-laden EEG spectrum after processing is shown in the middle panel. The clean EEG spectrum after processing is shown in the right panel for comparison.

An example file pre- and post-processing with HAPPILEE is shown in Fig. 8 to demonstrate the effectiveness of the pipeline. Clean 30 s segment of data pre- and post-processing is shown as well as an artifact-laden 30 s segment of data pre- and post-processing. The clean and artifact-laden power spectra are much more similar post-processing compared to pre-processing.

### 1.10 HAPPILEE outputs

HAPPILEE outputs include the processed EEG and the HAPPILEE processing reports. These outputs are generated in several folders that are located within the user-specified folder of files for processing. EEG files are saved out after several intermediate processing steps so that users can explore in-depth and visualize how those steps affected the EEG signal in their own datasets. The intermediate files are separated into folders based on the level of processing performed on the data and include: (1) data after filtering to 100 Hz and line-noise reduction, (2) data post-bad channel rejection (if selected), and (3) post-wavelet-thresholded data. If segmenting is enabled, HAPPILEE outputs one to two additional intermediate files: (5) post-segmented EEG data (always) and (6) interpolated data (if bad data interpolation is enabled). If segment rejection is selected, HAPPILEE saves the data post-segment rejection as well.

HAPPILEE outputs fully processed files that are suitable inputs for further analyses in one of several formats, selected by the user at the start of the HAPPILEE run, to increase compatibility with other software for data visualizations or statistical analyses. Options include mat, .set, and .txt formats. Alongside the fully processed data, HAPPILEE also outputs the HAPPE Data Quality Assessment Report and the HAPPE Pipeline Quality Assessment Report, each described in detail below. Finally, if HAPPILEE is run in the semi-automated setting, the software generates an image for each file containing the fully processed data's power spectrum.

## 2 HAPPILEE data quality assessment report

HAPPILEE generates a report table of descriptive statistics and data metrics for each EEG file in the batch in a single spreadsheet to aid in quickly and effectively evaluating data quality across participants within or across studies. The report table with all these metrics is provided as a .csv file in the “quality_assessment_outputs” folder generated during HAPPILEE. We describe each of these metrics below to facilitate their use to determine and report data quality (for an example data quality assessment report, see Table 6).

**Table 6:** Example HAPPILEE data quality assessment report for the 30 files in the example dataset.

### 2.1 File length in seconds

HAPPILEE outputs the length, in seconds, of each file prior to processing.

### 2.2 Number of user selected channels

HAPPILEE outputs the number of channels included for each file as determined by the user.

### 2.3 Number of good channels selected and percent of good channels selected

The number of channels contributing data (“good channels”) and the percentage of channels remaining following rejection of bad channels are provided.

### 2.4 Bad channel IDs

The identity of channels that are marked bad during the bad channel detection step and subsequently interpolated are provided. Users wishing to limit the amount of interpolated data in further analyses can easily identify channels for exclusion using this metric. Users may also reject files from further analysis on the basis of too high a percentage of bad channels.

### 2.5 Percent variance retained post-wavelet

The percent change of the variance of the signal following wavelet thresholding compared to before wavelet thresholding is provided for the user to evaluate how much data is retained following this step of artifact correction.

### 2.6 Channels interpolated per segment

Users that choose to perform bad data interpolation within segments (as in FASTER, Nolan et al. 2010) will be provided with the list of channels interpolated for each segment.

### 2.7 Number of segments pre-segment rejection and number of segments post-segment rejection

HAPPILEE reports the number of segments before segment rejection and post segment rejection (using joint probability, amplitude-based rejection, or both). If segment rejection is not enabled, these numbers are identical. If the user enabled segment rejection in HAPPILEE, they may evaluate the number of data segments remaining post-rejection for each file to identify any files that cannot contribute enough clean data to be included in further analyses (user discretion). The user may also easily tabulate the descriptive statistics for remaining segments to report in their manuscript's Methods section (e.g., the mean and standard deviation of the number of usable data segments per file in their study).

### 2.8 Percent of segments post-segment rejection

The percentage of segments that remain following segment rejection (using joint probability, amplitude-based rejection, or both) are provided for the user.

## 3 HAPPILEE pipeline quality assessment report

For each run, HAPPILEE additionally generates a report table of descriptive statistics and data metrics for each EEG file in the batch in a single spreadsheet to aid in quickly and effectively evaluating how well the *pipeline* performed across participants within or across studies. Note that these metrics may also be reported in manuscript methods sections as indicators of how data manipulations changed the signal during pre-processing. The report table with all these metrics is provided as a .csv file in the “quality_assessment_outputs” folder generated during HAPPILEE processing (for an example pipeline quality assessment report, see Tables 7 and 8).

**Table 7:** Example HAPPILEE pipeline quality assessment report with line noise removal metrics for the 30 files in the example dataset.

**Table 8:** Example HAPPILEE pipeline quality assessment report with waveleting metrics for the 30 files in the example dataset.

### 3.1 r pre/post linenoise processing

HAPPILEE automatically outputs cross-correlation values at and near the specified line noise frequency (correlation between data at each frequency before and after line noise processing). These cross-correlation values can be used to evaluate the performance of line noise attenuation, as the correlation pre- and post-line noise alogirthm should be lower at the specified frequency or frequencies, but not at the surrounding frequencies beyond 1 to 2 Hz (unless those are also specified by the user). HAPPILEE will automatically adjust which frequencies are reported depending on the user-identified line noise frequency. This metric can also be used to detect changes in how much line noise is present during the recordings (e.g., if generally cross-correlation values are high when study protocol is followed, indicating low line-noise removal from the data, but a staff member forgets and leaves their cell phone on the amplifier for several testing sessions, the degree of line noise removal for those files summarized by this metric could be used as a flag to check in on site compliance with acquisition protocols).

### 3.2 r pre/post wav-threshold

HAPPILEE automatically outputs the cross-correlation values before and after wavelet thresholding across all frequencies and specifically at 0.5, 1, 2, 5, 8, 12, 20, 30, 45, and 70 Hz. These specific frequencies were selected to cover all canonical frequency bands across the lifespan from delta through high-gamma as well as the low-frequencies retained in ERP analyses. These cross-correlation values can be used to evaluate the performance of waveleting on the data for each file. For example, if cross-correlation values are below 0.2 for all participants in the sample, the wavelet thresholding step has not progressed as intended (users are advised to first check their sampling rate in this case and visualize several raw data files). Note that this measure may also be used to exclude individual files from further analysis based on dramatic signal change during waveleting (indicating high degree of artifact), for example if the 8 Hz or all-data cross-correlations are below some threshold set by the user (e.g., 3 standard deviations from the median or mean, *r* values below 0.2).

Through these quality assessment reports, HAPPILEE aims to provide a rich, quantifiable, yet easily accessible way to effectively evaluate data quality for even very large datasets in the context of automated processing. Visual examination of each file is not required, although it is available. We also hope to encourage more rigorous reporting of data quality metrics in manuscripts by providing these outputs already tabulated and easily transformed into descriptive statistics for inclusion in reports. Users may also wish to include one or several of these metrics as continuous nuisance covariates in statistical analyses to better account for differences in data quality between files or verify whether there are statistically significant differences in data quality post-processing between study groups of interest.

Several metrics may also be useful in evaluating study progress remotely to efficiently track the integrity of the system and data collection protocols. For example, the r pre/post linenoise removal metric may indicate environmental or protocol deviations that cause significant increases in line noise in the data, and the Percent Good Channels Selected and Interpolated Channel ID metrics can be used to track whether the net/cap is being applied and checked for signal quality prior to paradigms or whether a channel (or channels) is in need of repair. For example, if the T6 electrode starts consistently returning bad data for a specific net/cap, it may need to be examined for repair. For further guidance about using the processing report metrics to evaluate data, users may consult the User Guide distributed with HAPPILEE software.

## 4 HAPPILEE compared to other low-density pre-processing approaches

The HAPPILEE pipeline uses wavelet-thresholding based artifact-correction methods to improve pre-processing capabilities for low-density EEG. Below we compare HAPPILEE's approach to two other common pre-processing methods, independent component analysis (ICA) for artifact correction, and voltage-thresholding segment rejection for artifact rejection, to justify the choice of wavelet-thresholding in HAPPILEE's pre-processing sequence.

### 4.1 HAPPILEE's wavelet-thresholding vs. ICA

Automated artifact correction approaches in high-density EEG pipelines to date have relied on independent component analysis (ICA) and wavelet thresholding methods instead as they can successfully remove artifact while retaining the entire length of the data file. ICA clusters data across electrodes into independent components that can segregate artifacts from neural data, while wavelet-thresholding parses data within frequency ranges using coefficients that can detect artifact fluctuations in either electrode data or independent components. ICA is included as an artifact rejection approach in many pipelines, including MADE (Debnath et al., 2020) and HAPPE 1.0 (Gabard-Durnam et al., 2018) software. Wavelet thresholding was also implemented in HAPPE 1.0 (Gabard-Durnam et al., 2018) as part of an initial wavelet-enhanced ICA (W-ICA) artifact rejection step (see Castellanos and Makarov 2006 for details).

Importantly, prior literature suggests that ICA is not an optimal artifact rejection tool for low-density EEG configurations, as the number of channels determines the number of independent components to be generated, and many low-density configurations have too few channels to adequately segregate artifact from neural components sufficiently. For example, Troller-Renfree and colleagues have indicated through their MiniMADE pipeline that ICA performs poorly on their low-density data to remove artifacts (the MiniMADE pipeline instead uses threshold-based rejection to remove eye blinks) (Troller-Renfree et al., 2021). ICA has also failed to remove classes of artifact like line noise in low-density data (Xue et al., 2006). In contrast, wavelet-thresholding operates on each EEG channel separately, and thus should have density-independent artifact-correction performance. Indeed, wavelet thresholding has been found to outperform denoising methods that could apply to low-density data, including Empirical Mode Decomposition, Kalman filtering (Salis et al., 2013), and ICA. Specifically, prior studies have found that wavelet thresholding outperforms ICA as an artifact removal approach on data with fourteen channels (Bajaj et al., 2020). Krishnaveni and colleagues also found that waveleting performs better than ICA (using the JADE algorithm) in removing eye blinks in the low frequency range while preserving brain signal in high frequencies (Krishnaveni et al., 2006).

Wavelet thresholding has several additional advantages beyond channel-density independent performance when compared to other artifact correction and rejection approaches. First, wavelet-thresholding can be applied down to the level of single-channel EEG recordings, as it has no minimum channel number to run effectively. Additionally, wavelet thresholding corrects artifact without removing discrete timepoints from the EEG recording, an issue that is inherent to manual artifact rejection where whole EEG segments are rejected (and so data from clean channels during that segment are sacrificed). Relatedly, wavelet thresholding can detect and remove isolated and non-stereotyped artifacts easily without removing neural data from other points in time or other channels, whereas ICA must reject an entire timeseries (the component) to remove the artifact, even if it occurs relatively rarely over the recording (or ICA must be paired with initial segment rejection, which sacrifices good data from other electrodes during those artifact-contaminated segments). Wavelet thresholding is also computationally more efficient than either ICA or manual inspection (with results that are perfectly reproducible each time the method is applied to the same dataset). All of these features make wavelet thresholding appealing as a strategy for removing artifact from low-density EEG layouts.

In addition to the prior literature and conceptual reasons provided so far in favor of wavelet-thresholding as an artifact-correction strategy for low-density recordings, we ran a series of empirical tests to provide evidence for our decision to implement wavelet thresholding, rather than ICA, as our artifact correction method. In order to test ICA's performance as a function of channel density, we ran ICA under two conditions: on a subset of 5 channels (F3 F4 FZ C3 C4) and on 12 channels downsampled to the same 5 channels following ICA correction. We predicted that ICA would perform differently as a result of varying the number of channels run on artifact correction for the two datasets. Confirming our prediction, we found that ICA run on five channels rejected all independent components on a total of five files, while ICA run on twelve channels did so for only two files. That is, processing performance differed as a function of channel density during ICA.

**Fig. 9:**
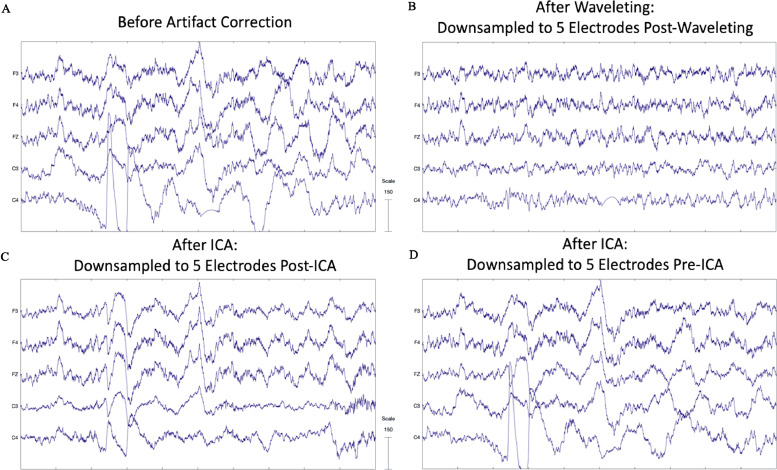
EEG signal before artifact correction (A), after waveleting (B) and after ICA (C) when the file is downsampled to 5 electrodes following artifact correction, and after ICA when the file is downsampled to 5 electrodes before artifact correction (D). All scales are in microvolts.

Moreover, downsampling before vs. after artifact correction led to differing performances of ICA, as measured by percent variance retained following ICA (t(8) = 2.61, *p* = 0.03); see Table 9 below. Further, these findings are supported by clear visual differences between the post-artifact corrected signal when downsampling occurs before vs. after ICA (Fig. 9). Notably, bad channel detection was not run for this assessment, but these findings raise concerns that removing bad channels and thus changing the channel density for ICA across files within a dataset would vary ICA performance within that dataset. Meanwhile, running the same files under the above conditions through waveleting made no difference as the artifact correction runs within each channel independently (and thus is agnostic to channel density differences within or across files). That is, wavelet thresholding for artifact correction within a dataset is never channel-density dependent, so correction will operate similarly if a file has many, few, or no bad channels flagged.

**Table 9:** Percent variance retained following ICA and waveleting for files with 5 channels (F3 F4 FZ C3 C4) and files with 12 channels downsampled to the same 5 channels.

**Fig. 10:**
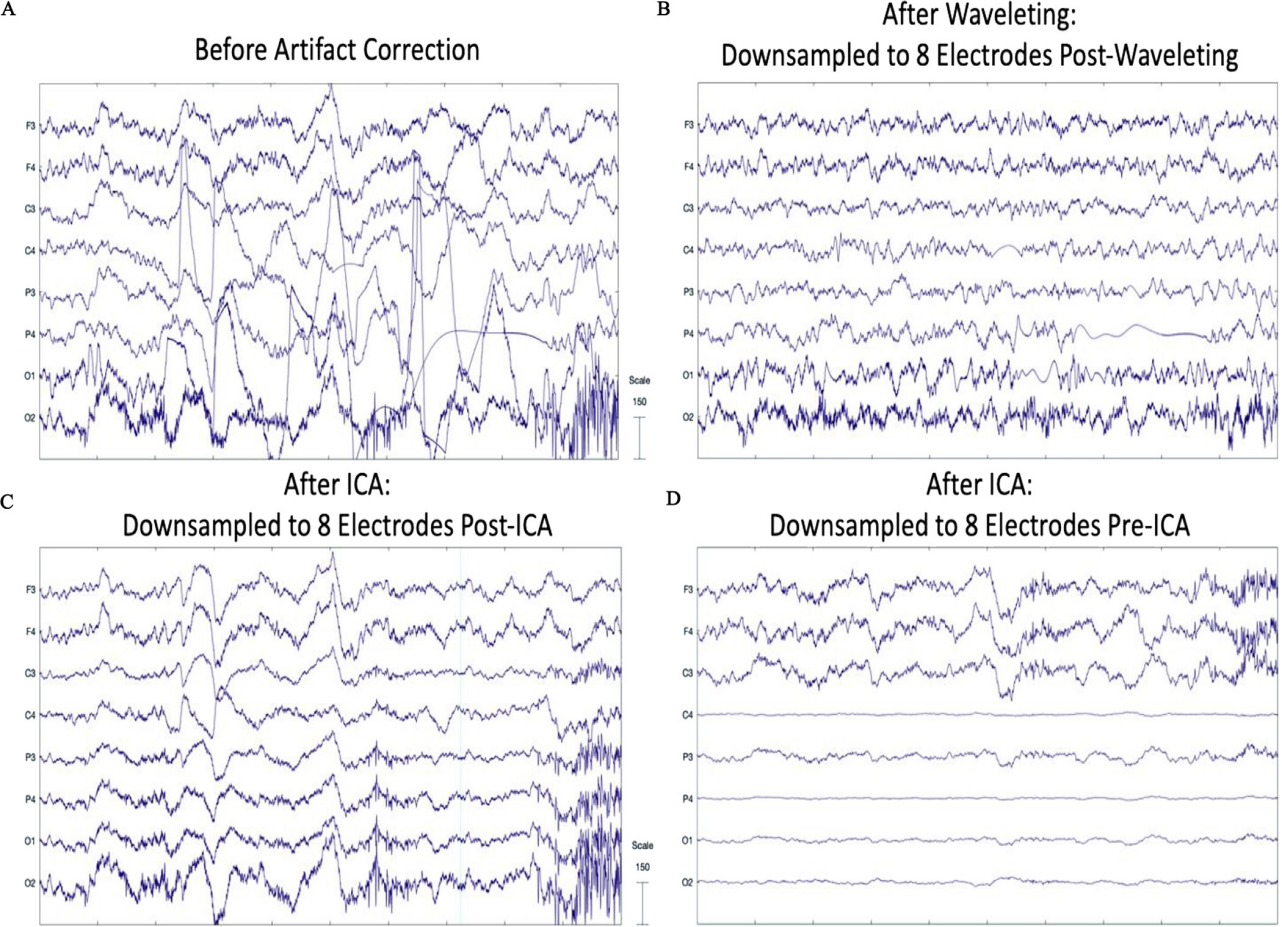
EEG signal before artifact correction (A), after waveleting (B) and after ICA (C) when the file is downsampled to 8 electrodes following artifact correction, and after ICA when the file is downsampled to 8 electrodes before artifact correction (D). All scales are in microvolts.

As one additional test of ICA's performance on low density datasets under conditions where bad channels could more commonly alter channel density, we ran ICA on a subset of 8 channels (F3 F4 C3 C4 P3 P4 O1 O2) and on 12 channels downsampled to the same 8 channels following ICA correction (several files in the BEIP dataset had 3–4 bad channels resulting in ICA on 8–9 good channels instead of the full 12 channels). Once again, as predicted, we found that downsampling before vs. after artifact correction led to differences in the performance of ICA, as ICA run on 8 channels removed all components on four files, while downsampling to 8 channels rejected all components on only two files. For the subset of files retained by both processing runs, although there were not significant differences between processing conditions measured by percent variance retained following ICA (*p* > 0.05), several individual files experienced dramatic changes in variance retained as a function of channel density (e.g., 13 vs 31%, 60 vs 40%); see Table 10 below. Once again, there are clear visual differences between the post-artifact corrected signal when downsampling occurs before vs. after ICA (Fig. 10; significant signal loss in multiple channels observable in the case of downsampling before running ICA).

**Table 10:** Percent variance retained following ICA and waveleting for files with 8 channels (F3 F4 C3 C4 P3 P4 O1 O2) and files with 12 channels downsampled to the same 8 channels.

This pattern may indicate inconsistent ICA results across these channel densities. Again, as expected, the percent variance retained for waveleting and files rejected were identical for 8 channels compared to 12 channels downsampled to 8 channels. These results together further solidify our decision to use waveleting instead of ICA to ensure consistent performance across files regardless of differences in bad channel number with low density data in HAPPILEE.

**Fig. 11:**
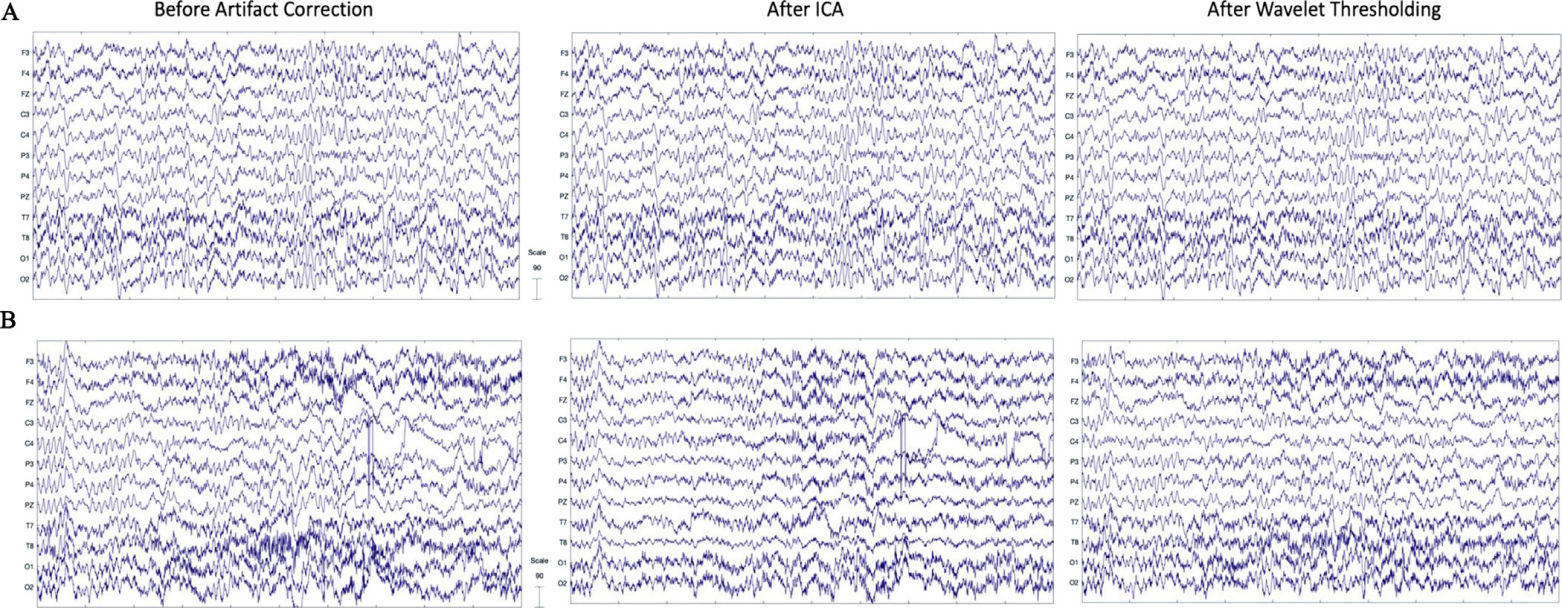
EEG signal before artifact correction, after ICA, and after wavelet thresholding. Two files from the same participant in the example dataset are shown with 10 s of data extracted from the clean 30 s segment (A) and artifact-laden 30 s segment (B). The EEG signal before artifact correction is shown in the left panel. The EEG signal after ICA is shown in the middle panel. The EEG signal after wavelet thresholding is shown in the right panel. All scales are in microvolts.

Even with the complete set of channels in a low-density context, ICA underperformed in artifact correction relative to wavelet-thresholding visually (Fig. 11). For example, ICA and wavelet thresholding perform similarly on the clean segment where there is very little artifact to correct, but high amplitude artifact clearly remains in the artifact laden segment after it is run through ICA (Fig. 11). Meanwhile, wavelet thresholding effectively removes the high amplitude artifact.

Beyond the inconsistency of ICA's performance as a function of channel density with low density datasets, ICA is also far less computationally efficient than waveleting, reflected both mathematically (e.g., complexity of a learning algorithm vs. wavelet function) and in differences in processing time on the same machine. When run on twelve full length BEIP files averaging roughly three and a half minutes in length and containing 12 channels, ICA was much slower to complete than wavelet-thresholding. ICA averaged 165 s per file while waveleting averaged 1 s per file for artifact correction. Both the prior literature and these additional assessments suggest that waveleting-based approaches will provide the more efficient and consistent performance relative to ICA across the range of low-density layouts considered here.

We do note that how wavelet-thresholding (spatially-independent approach) and spatial filtering approaches (e.g. ICA, PCA) perform relative to each other in correcting artifacts in EEG may depend on several contextual parameters that the field has yet to systematically explore. For example, the inter-channel distance, total spatial coverage on the head across channels, number of channels, and type of artifact signal may all affect how ICA performs relative to wavelet-thresholding. Here, channels were spatially distributed across the scalp and a variety of artifact types were included but not compared directly to each other. Further research is required to systematically quantify how these factors impact artifact correction performance across layout configurations and age.

### 4.2 HAPPILEE's wavelet-thresholding correction vs. segment rejection

Another option for addressing artifact involves no artifact-correction steps (e.g., wavelet-thresholding, ICA), instead just artifact rejection through segment/trial rejection. Although no manipulations are performed on the retained data segments in the “segment rejection only” approach, there is loss of good data from channels without artifact in the segments that are rejected. Here we examined whether the inclusion of wavelet thresholding reduced the need to reject segments or trials of data downstream in the pipeline. To do so, we compared segment rejection rates in both the simulated and real EEG datasets.

With respect to the simulated data that had adult eye-blink and other developmental artifacts added, an automated amplitude-based voltage threshold (−100 to 100 μV threshold) was used for segment rejection comparisons. Only the wavelet thresholding approach retained all trials of simulated signal (Table 11) and appeared (based on visual inspection) to have removed all blinks and most of the other artifacts. In contrast, when using artifact rejection only, epochs exceeding the voltage threshold were removed, including some epochs with blinks, but not all. A total of 116 epochs were contaminated with blink artifact, but only 17 epochs were removed with voltage threshold artifact rejection when additional artifact was included in the simulated data (in a separate test, not reported in detail here, we ran the data with just blink artifact included and 107/116 epochs with blinks were removed with voltage threshold artifact rejection). Visual inspection of the data suggested that some of the blinks in noisier epochs were retained when subjected to only artifact-rejection thresholding because the noise stemming from muscle activity included negative deflections that lowered the amplitude of the blinks, which caused the blink artifacts to fall within our acceptable voltage threshold range. Of note, some muscle activity still remained in the simulated data in all pre-processing conditions in this test. These simulated data results strongly support the use of HAPPILEE's wavelet-thresholding as an artifact-correction approach prior to segment rejection to improve both artifact removal and segment retention.

**Table 11:** Performance of two pre-processing sequences on simulated EEG data and real EEG data.

To further evaluate the performance of segment retention only compared to wavelet-thresholding, the real EEG data in the BEIP study was leveraged with manual segment rejection. Manual segment rejection here addressed the effect observed above in the simulated data where some artifacts in the segment rejection only approach passed through automated voltage thresholds when combined with other artifacts (as often occurs in real EEG data). Specifically, manual segment rejection rates were compared in 14 files of the BEIP dataset that were processed twice, once with and once without wavelet-thresholding on the data (mean number of segments before rejection = 105.6). The mean number of clean segments retained after manual rejection on post-waveleted data (85.7 segments) was significantly higher than the mean number of clean segments retained without wavelet thresholding (62.6 segments; t(18) = −9.07, *p* = 0.00000004, Table 11). That is, artifact-correction via wavelet-thresholding improved segment retention by 37% relative to no artifact-correction before segment rejection in real EEG data. This pattern of results across comparisons with ICA and artifact rejection via voltage-thresholding provides consistent evidence in support of using wavelet-thresholding for artifact-correction in pre-processing EEG data in low-density contexts.

## 5 Conclusion

The field of cognitive neuroscience has been rapidly moving toward the use of automated EEG pre-processing pipelines that make use of contemporary artifact correction and rejection approaches like wavelet-thresholding and ICA as effective, efficient, standardized alternatives to subjective and labor-intensive manual pre-processing. Here we provide a solution suitable for lower-density layouts from approximately 32 channels down to single channel EEG with the current automated pipeline, HAPPILEE. HAPPILEE supports processing resting-state and task-related EEG, as well as ERP data (see Monachino et al. 2021 for details on optimization for ERP analyses). HAPPILEE is suitable for configurations with any number of channels, though it may perform best on data with 1 to around 32 channels (those with higher-density configurations may consider pipelines optimized for high-density data, including the companion HAPINNES or HAPPE+ER (Monachino et al., 2021) pipelines within HAPPE 2.0 software.

There are several limitations to HAPPLIEE that should also be considered. First, HAPPILEE was optimized and validated using developmental EEG and simulated signals, so it remains to be validated in adult EEG data or other populations. Though the authors do not anticipate specific reasons HAPPILEE would not perform well in other populations, and have run HAPPILEE in adult EEG data themselves, researchers with EEG data from populations not validated in this manuscript are encouraged to carefully verify performance themselves before using HAPPILEE. Second, bad channel detection was tested on a dataset with twelve channels. As a result, those working with layouts with substantially fewer electrodes may consider verifying for themselves that the default settings work sufficiently well for their datasets. Alternatively, the bad channel detection step is optional, so if it is unsuitable or is not desired for a dataset, the user may opt-out of this step of the pipeline. Furthermore, the appropriate amplitude threshold for performing segment rejection by amplitude will vary across datasets collected on different ages or systems and should be verified through visual inspection of several files (via running HAPPILEE in the semi-automated setting with visualizations). Lastly, HAPPILEE was optimized using a single, gel-based (low-impedance) system (James Long) given the data available to the researchers, but others should independently verify performance on the other systems and recording contexts to confirm compatibility with HAPPILEE. Although the authors do not currently foresee any difference in performance between datasets collected in laboratory environments (i.e., the current optimization dataset) and datasets collected in the home or clinics, further testing will be necessary in order to fully verify its efficacy on data collected in non-laboratory settings.

The HAPPILEE pipeline is freely available as part of the HAPPE software (first released with HAPPE version 2.0), covered under the terms of the GNU General Public License (version 3) (Free Software Foundation, 2007). HAPPILEE's sequence of processing steps are automatically triggered within HAPPE 2.0+ software when the user indicates they have data with fewer than 32 channels. HAPPILEE may be accessed at: https://github.com/PINE-Lab/HAPPE. The HAPPE 2.0+ software download includes a user guide to aid in the set-up and implementation of the pipeline. The subset of BEIP EEG data used to optimize the HAPPILEE pipeline, including the files used in the clean vs. artifact and artifact addition approaches and simulated signals are publicly available at: https://zenodo.org/record/5088346 (Lopez et al., 2021).

## Funding

This project was supported via a grant from the Bill and Melinda Gates Foundation to LGD.

## Code and data availability

All code used in the HAPPILEE pipeline is freely available under the terms of the GNU General Public License at: https://github.com/PINE-Lab/HAPPE.

All EEG and simulated EEG data used to optimize the HAPPILEE pipeline in this manuscript are freely available as a dataset through Zenodo as Lopez et al., 2021: https://zenodo.org/record/5,088,346.

## CRediT authorship contribution statement

**K.L. Lopez:** Data curation, Formal analysis, Methodology, Software, Validation, Writing – original draft, Writing – review & editing. **A.D. Monachino:** Methodology, Software, Validation, Writing – original draft, Writing – review & editing. **S. Morales:** Data curation, Formal analysis, Validation, Writing – original draft, Writing – review & editing. **S.C. Leach:** Data curation, Formal analysis, Validation, Writing – original draft, Writing – review & editing. **M.E. Bowers:** Data curation, Formal analysis, Validation, Writing – original draft, Writing – review & editing. **L.J. Gabard-Durnam:** Conceptualization, Funding acquisition, Methodology, Project administration, Software, Supervision, Validation, Writing – original draft, Writing – review & editing.

## Appendix Supplementary materials

Image, application 1
