# Supplementary material for: HAPPILEE: HAPPE In Low Electrode Electroencephalography, a standardized pre-processing software for lower density recordings
Source: Neuroimage. 2022 Oct 15;260:119390. doi: 10.1016/j.neuroimage.2022.119390 (PMC9395507; doi:10.1016/j.neuroimage.2022.119390)
Supplement: Supplementary file 1 [file mmc1.docx]

**Supplemental File 1: Types of artifact isolated and added into EEG data during validation**


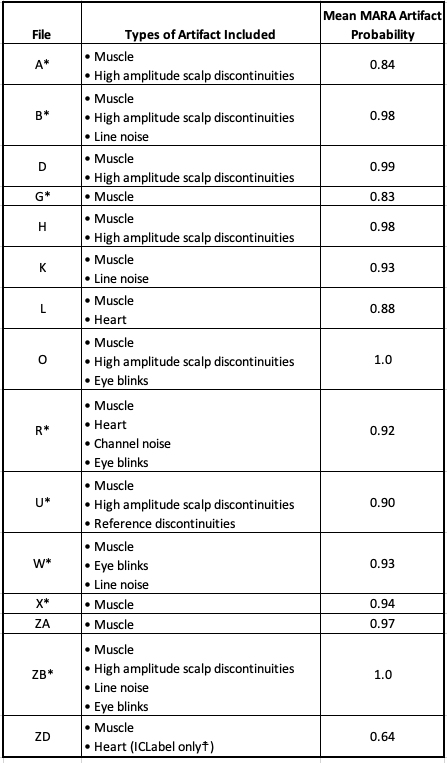


[*denotes artifact used in simulated data; all files used in artifact-added approach

☨Heart artifact not detected using MARA but marked by ICLabel and confirmed visually

Simulated data additionally contains reliable adult eye blink artifact not included in this table of developmental artifacts]
